# Introduced and native vertebrates in pink-footed shearwater (*Ardenna creatopus*) breeding colonies in Chile

**DOI:** 10.1371/journal.pone.0254416

**Published:** 2021-07-29

**Authors:** Ryan David Carle, Abram B. Fleishman, Tiare Varela, Pablo Manríquez Angulo, Guillermo De Rodt, Peter Hodum, Valentina Colodro, Verónica López, Héctor Gutiérrez-Guzmán

**Affiliations:** 1 Oikonos Ecosystem Knowledge, San Juan Bautista, Robinson Crusoe Island, Valparaíso Region, Chile; 2 Conservation Metrics, Inc., Santa Cruz, California, United States of America; 3 University of Puget Sound, Tacoma, Washington, United States of America; MARE – Marine and Environmental Sciences Centre, PORTUGAL

## Abstract

Biodiversity conservation planning requires accurate, current information about species status and threats. Although introduced mammals are the greatest threat to seabirds globally, data on introduced species is lacking for many seabird breeding islands. To inform conservation planning, we used trail cameras to document the presence, relative abundance, and seasonal and diel attendance of introduced and native vertebrates within pink-footed shearwater (*Ardenna creatopus*) breeding colonies on Isla Mocha (five colonies, 2015–2020) and Isla Robinson Crusoe (Juan Fernández Archipelago), Chile (one colony, 2019–2020). The most commonly detected species were pink-footed shearwaters and introduced rats (*Rattus* spp.) on Isla Mocha, and European rabbits (*Oryctolagus cuniculus*) and pink-footed shearwaters on Isla Robinson Crusoe. Introduced mammals observed, in order of greatest catch-per-unit-effort, were rats, cats (*Felis catus*), dogs (*Canis lupus familiaris*), and European hares (*Lepus europaeus*) on Isla Mocha and European rabbits, cats, cattle (*Bos taurus*), rats, dogs, mice (*Mus musculus*), and southern coati (*Nasua nasua*) on Isla Robinson Crusoe. Especially noteworthy results for pink-footed shearwater conservation were the presence of cats during all monitoring months in shearwater colonies on both islands, that catch-per-unit-effort of rabbits was greater than shearwaters on Isla Robinson Crusoe, and that rats were the most observed vertebrates after shearwaters on Isla Mocha. Pink-footed shearwaters were regularly present on the islands from October through May. Presence and relative catch-per-unit-effort of pink-footed shearwaters qualitatively matched the species’ known breeding phenology. The regular presence and temporal overlap with shearwaters of cats, rats, rabbits, and cattle within shearwater colonies, coupled with the irregular presence of dogs, coati, hares, and mice, indicated a serious conservation threat for pink-footed shearwaters and other native insular fauna and flora. Finally, our study provides a widely applicable model for analysis of multi-year trail camera data collected with unstandardized settings.

## Introduction

Biodiversity conservation planning requires accurate, current information about species status and threats [1, 2]. Considerable conservation planning and prioritization have been focused on seabirds [3, 4], one of the most threatened bird taxa in the world [5]. At a global level, these planning efforts have identified that introduced mammals such as feral cats (*Felis catus*) and rats (*Rattus* spp.) on island breeding colonies are the greatest threat to seabirds [5]. Consequently, seabird conservation actions often focus on identifying and implementing high priority eradications of introduced vertebrates from islands [2, 6]. Despite global attention toward these efforts, 20% of known seabird breeding islands lack data of any kind on introduced species [3], as do 20% of all islands worldwide [7]. Species introduction and extirpation are dynamic processes, and many islands lack biosecurity protocols, meaning that data on the presence of introduced species must be regularly updated. Though there are systematic databases for storing data on introduced species, there is no comprehensive worldwide system for collection of such data [8]. Thus, the availability of information about introduced species on a given island depends on factors including accessibility (both in a global geographical sense and a local topographical and infrastructural sense), global and local perception of conservation need and subsequent funding, and engagement with knowledge of local residents on inhabited islands. Additionally, for evaluating the threat of a given introduced species on the native ecosystem, basic information on species presence/absence is most useful when coupled with information about that species’ relative or absolute abundance. It is also important to understand the presence, relative abundance and attendance patterns of introduced species at the scale when and where they are most likely to impact threatened native species. In the case of seabirds breeding in discrete areas of large islands, this scale is within specific breeding colonies, rather than island-wide. Likewise, information about the relative abundance and temporal attendance of introduced species is useful for assessing the likelihood and severity of interactions with native species.

We used trail cameras to investigate the assemblage, relative abundance, and seasonal and diel attendance of introduced and native vertebrates on Isla Mocha and Isla Robinson Crusoe (hereafter IRC), Chile, two of three islands where pink-footed shearwaters (*Ardenna creatopus*) are known to breed worldwide [9, 10]. The world breeding population of pink-footed shearwaters is currently estimated to be approximately 60,000 individuals [11, 12]. This population estimate is currently being revised, but studies have consistently concluded that at least 70% of the world population nests on Isla Mocha, and the remainder nests on the Juan Fernández Archipelago on IRC and Isla Santa Clara (≤20% and ≤10% of the world population, respectively [10, 11, 13]. During the non-breeding period, pink-footed shearwaters leave breeding islands and migrate northward as far as Canadian waters [14]. The pink-footed shearwater is listed by the IUCN as Vulnerable [15], and by Chile and Canada as Endangered [16, 17], due to its restricted breeding range, relatively small population size, and threats including introduced mammals on breeding colonies [18] and mortality from fisheries bycatch [12, 14].

Both Isla Mocha and IRC are inhabited by humans, which is a strong predictor of the presence of introduced species on islands [7]. On Isla Mocha, notions of whether some species are native are complicated because the island has been inhabited by humans for >3,000 years [19, 20]. We defined “introduced species” as those introduced post-European contact. Introduced mammals with self-sustaining wild populations reported on Isla Mocha are cats, dogs (*Canis lupus familiaris*), black and Norway rats (*Rattus rattus* and *R*. *norvegicus* respectively), and house mice (*Mus musculus*) [13, 21–23]. Five rodent species are native to Isla Mocha [22, 24]. It is unclear whether the pudú, a small deer, should be considered native to Isla Mocha; the archaeological record indicates it was brought to the island by indigenous people [19], but it may have been extirpated and reintroduced in recent decades (V. López, pers. obs.). Isla Mocha also has managed populations of cattle (*Bos taurus*), horses (*Equus caballus*), and other livestock that are limited to a low elevation coastal plain. On IRC, which has been inhabited by humans only post-European contact, introduced species with self-sustaining wild populations reported are cats, dogs, southern coati (*Nasua nasua*), goats (*Capra hircus*), European rabbits (*Oryctolagus cuniculus)*, black and Norway rats and house mice [21, 25–28]. IRC also has managed populations of cattle, horses, mules (*E*. *asinus* x *E*. *caballus*) that range in wildlands [25, 26]. Primary and secondary sources for introduced species observations for each island are listed in S1 Table. Neither Isla Mocha nor IRC has a biosecurity system, and new animal introductions are possible through regular human travel between the islands and the mainland.

The goal of our study was to better inform conservation planning for pink-footed Shearwaters and other native species on Isla Mocha and IRC. Although the assemblage of introduced mammals on Isla Mocha and IRC is documented in published literature, we sought to advance our knowledge of the nature and extent of the threat of introduced species on these islands in several ways. First, though there are published records of the introduced species assemblages on both islands, most records were secondary citations of a limited number of primary sources (S1 Table). There was only one study documenting the introduced fauna on Isla Mocha with primary observations [22], and two for IRC [25, 27] (as well as a possible third we were unable to access [29]). All studies with primary observations were ≥19 years old, and three were >40 years old at the time of our study, suggesting that our understanding of the species present was outdated. Thus, we sought to update our knowledge of the assemblage of introduced species on these islands, and consolidate this knowledge in one accessible study providing a baseline for future comparisons. We also sought to advance from a simple list of introduced animals present on each island by quantifying for the first time the presence, relative abundance, and seasonal and diel attendance of introduced species within pink-footed shearwater breeding colonies. Finally, for Isla Mocha, a further objective was to develop methods for analysis of multi-year trail camera monitoring data collected with unstandardized methods, an application widely applicable to conservation of other species and ecosystems.

## Materials and methods

Animal care and use was reviewed and approved by the BioBío Region of Corporacion Nacional Forestal (CONAF), Chilean Departamento de Areas Silvestres Protegidas of Chile. Bring a purely observational study, further steps were not required to ameliorate suffering nor was review required by other agencies.

To quantify the presence and seasonal and diel attendance of animals on Isla Mocha and IRC, we used motion-triggered trail cameras with built-in infrared illuminators for capturing night images. We set cameras to capture images (both photos and videos) 24 hours a day. Details of the methods and duration of the study differed by island.

### Isla Mocha

We deployed trail cameras at five pink-footed shearwater colonies on Isla Mocha during 2015–2020 (S2 Table). Isla Mocha (47.82 km^2^, 38.383°S, 73.900°W) is 34.2 km offshore of the mainland of south-central Chile. A densely forested central mountain range on Isla Mocha rises to 390 m elevation and is protected as a Chilean National Reserve. Approximately 650 people live in decentralized homesteads on the coastal plain skirting the mountains. Pink-footed shearwaters nest on steep slopes in the mountain above approximately ≥200 m elevation [11, 13].

We monitored colonies in forested areas at 200–350 m elevation and with relatively large numbers of shearwater nests (>100 burrows). Each monitored colony was approximately 1–2 hectares in size. Monitored colonies were spatially separated by >2 km, with one exception in which two colonies were 0.9 km apart. Habitat features and topography were qualitatively similar in the five colonies, in forested areas on or near sleep slopes near the tops of ridges. One colony was located along a hiking trail popular with tourists (Colony 1), one colony was located along a trail mainly used by local people (Colony 3), and three colonies were in places without trails and not typically visited by humans other than researchers (Colonies 2,4, and 5). All colonies were ≤1km from, but 200–300 m above, the nearest human settlements.

Within each colony, we deployed one to four cameras annually. Camera deployments dates varied, but the most consistent deployment months were from December to June, with occasional deployments in October, November, and July (S1 Fig). Deployments were timed to be during the pink-footed shearwater breeding season (November-May) [10]. We changed the positions of cameras within colonies season to season, but left cameras in fixed locations within monitoring seasons. All cameras were separated by at least 5 and at most 40 m, with non-overlapping views focused on different parts of the breeding colony. We did not measure burrow densities at camera deployment locations, but the average and maximum burrow densities of randomly selected 5 m radius plots containing burrows (n = 24 plots) in an unrelated study on Isla Mocha were 0.04 ± 0.03 SD and 0.11 burrows per m^2^, respectively (S2 Table). Cameras were 50–80 cm above the ground and focused on areas with pink-footed shearwater burrows. Vegetation at all camera locations was composed of forest with mature trees, relatively closed canopy, and an open understory with sparse or no vegetation, such that tree trunks might regularly obscure views of animals but understory vegetation rarely did so. We used various models of Bushnell, Reconyx, and Browning brand trail cameras, set to take either photos or videos. When three or four cameras were deployed in a colony, we set two to take photos and the others to take video; when fewer cameras were deployed in a colony, we used various combinations of photo and video settings. We did not document camera settings during the study, so we examined the data to retroactively determine the settings. Most deployments had a 1-second delay between photos, though some deployments had 5, 10, 30, 90, or 300 second delays between consecutive motion-triggers. Cameras taking video recorded for 15 seconds, with a 1-second delay after recording before taking a new video.

### Isla Robinson Crusoe

We deployed trail cameras at one pink-footed shearwater colony, Piedra Agujereada (hereafter PAG), on IRC during 2019–2020. IRC (48.0 km^2^, 33.640°S, 78.833°W) is located 660 km off the South American mainland. Ninety-six percent of IRC is protected as a Chilean National Park. A human community of approximately 1,000 residents lives in San Juan Bautista, the only town on IRC. The island’s topography, volcanic in origin, is rugged and steep. Human settlement of IRC began in 1591 with post-European contact colonization; there is no evidence of prehistoric human occupation [30]. Many formerly forested areas were converted to grassland or barren soil by deforestation and grazing [26, 31]. Pink-footed shearwater colonies on IRC are located from sea level to 300 m on moderate to steep slopes [18].

The PAG breeding colony is located in northeast IRC on a steep (~30–40°) southeast-facing slope ranging from 100–300 m elevation. The area has been deforested and vegetation is characterized by non-native grasses and low-growing forbs [32]. PAG is one of the two largest pink-footed shearwater colonies on IRC, with approximately 2,000 breeding pairs in a 12.3 hectare area [18, 32]. PAG is approximately 2.2 km straight-line distance, and 4.6 km by trail, from San Juan Bautista. We monitored PAG because of its accessibility, relatively large size, and plans to install a mammal exclusion fence at this site. A cattle exclusion fence, built in 2011, protected approximately half the area of PAG from cattle trampling and herbivory, but did not exclude other introduced species [32]. During late 2020 (post-study) this fence was upgraded to also exclude rabbits, cats, dogs, and coati.

We deployed three trail cameras at PAG from February 2019 to February 2020 and a fourth from January 2019 to December 2019 (S1 Fig). Most images were collected by Browning Strike Force Pro trail cameras (model BTC5HDP; Prometheus Group, LLC). However, after camera malfunctions we replaced one camera with a Browning Strike Force Pro XD (model BTC-5PXD) and one with a Reconyx Hyperfire Semi-covert IR (model HC500). Cameras were located inside the cattle exclusion fence, at least 50 and at most 115 m apart in areas with burrow densities of 0.10–0.15 burrows per m^2^ in 10 m radius circular plots around the camera location. Cameras were 115–143 cm off the ground and were focused perpendicular to the angle of the slope. Vegetation at all camera locations was composed mainly of grasses and forbs ≤30 cm in height [32]. Camera views did not overlap and camera positions were consistent the entire year. Distances between cameras at PAG were greater than those on Isla Mocha primarily because PAG is a large, contiguous breeding colony, whereas the colonies on Isla Mocha had smaller areas. Our goal was to cover distinct areas of the entire PAG colony with a camera each, whereas on the much smaller Isla Mocha colonies, one to four non-overlapping cameras covered much of the colony area. We set cameras to take 5 or 6 image bursts when the camera was motion-triggered, though some triggers resulted in 8- and 12-image bursts. We set cameras to pause for 5 minutes after a burst before another event could be triggered. We attempted to keep the exact frame captured by the cameras consistent, but cameras positions were often changed slightly by strong winds and/or by cattle pushing camera poles.

### Data generation

For both islands, we manually reviewed each photo and video and identified all vertebrates to species. Exceptions were introduced rats, categorized as *Rattus* spp., native rodents on Isla Mocha, categorized as *Rodentia* spp., and “unknown animal” on IRC for unidentifiable animals (e.g., eye shine or blurry night images). In describing methods and results, we refer to all categories of identified animals as “species” for understandability. For each photo or video, we recorded the number of individuals of each species, the date, and time to the second. We collapsed the dataset by 1 hour intervals because image contents in multi-image bursts were auto-correlated and because individual animals could not be identified. For each species in each hour, we used the highest single-image count as the count for that hour.

To accurately measure survey effort, we inserted zeros into the dataset as follows: 1) if an hour contained only “empty” images, we counted that hour as a zero for all species; 2) if an hour did not have a given species in it, we counted that hour as a zero for that species; 3) if an hour period passed with no camera triggers, we counted that hour as a zero for all species. We assumed that a camera was functioning the entire date if it was triggered on that date. If a camera was not triggered on a given date, we assumed the camera was working if less than seven days elapsed between the last photo of the previous event and the first photo of the next event (see S1 Fig for depiction of survey effort accounting for camera failures).

We calculated hourly catch per unit effort (CPUE), defined as the maximum number of individuals of each species recorded per camera per hour (using only the single image in each hour with the highest count of individuals of each species to avoid double counting the same individuals) [33]. We then averaged the hourly CPUE of each camera by day, and averaged the daily averages across cameras, to obtain a single average hourly CPUE for each species on each island. The resulting average hourly CPUE values were often very small, so to more easily visualize the results we created a 30-day mean CPUE for each species by multiplying the average hourly CPUE by 720 (24 hours*30 days). We did not account for known diurnal/nocturnal species behaviors, or detectability of species in day vs. night in our calculations of hourly CPUE, and report a standard hourly CPUE based on 24 hours of the day. We did not attempt to quantify absolute abundance or density of animals, and CPUE values are a relative, island-specific metric of how often species were perceived by trail cameras. We also present seasonal and diel attendance for each species by island, for which we created a species-specific relative hourly CPUE metric. We divided each species’ hourly CPUE by the maximum recorded hourly CPUE for that species on each island to standardize the scale of relative CPUE (to a proportion with a maximum of 1.0) to facilitate attendance pattern comparisons. We averaged hourly results by day to examine seasonal attendance levels, and by hour of the day to examine diel attendance levels. This unitless relative CPUE metric can be used to compare seasonal and diel attendance across islands, but does not reflect actual magnitude or relative abundance of species because the maximum CPUEs were species- and island-specific. For both islands, we compared the pink-footed shearwater CPUE results to the known breeding phenology of the species. We report means ± SD unless otherwise noted. We summarized and analyzed data using R version 4.0.3 [34].

One limitation of this study was that trail camera monitoring was unstandardized on Isla Mocha. The colonies monitored and general focus of cameras on shearwater burrowing areas were consistent from year to year on Isla Mocha, but camera settings, number of cameras, types of images collected, deployment length, and exact camera locations were not. Because of the unstandardized nature of the Isla Mocha dataset, we developed analytical methods (described previously) to retro-actively standardize the data as much as possible, while attempting not to over-apply the data to comparisons for which more standardized data would be necessary. Thus, we pooled results from the five monitored colonies on Isla Mocha and did not attempt to compare metrics among them because they were monitored with differing efforts and methods. We do discuss differences in the basic presence/absence of species among colonies on Isla Mocha.

A related limitation of the study is that trail camera methods were standardized on IRC and therefore differed from methods on Isla Mocha, such that the camera settings, positioning, and study duration differed between islands. We could therefore not make direct comparisons of the relative abundance of species on the two islands, and results for each species should be interpreted as island-specific and comparable only to other species on that island. An exception is the relative seasonal and diel CPUE metric scaled to the proportion of the maximum CPUE on each island; this metric can be used to compare attendance of species across islands, with the caveat that the maximum CPUE differed on each island.

## Results

### Isla Mocha

**Fig 1:**
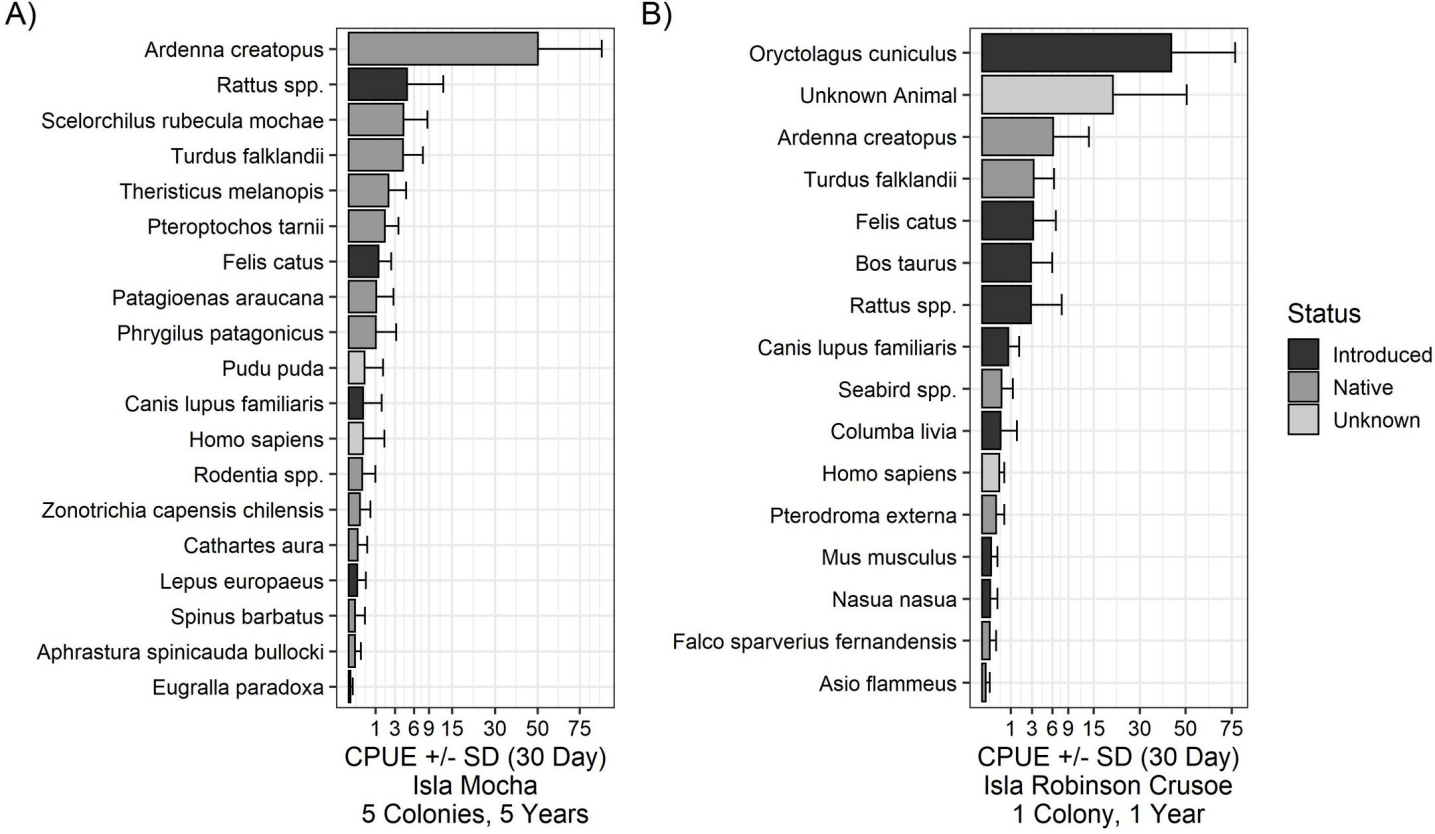
Mean 30-day catch per unit effort (CPUE) ± SD for each species on Isla Mocha (A) and Isla Robinson Crusoe (B), Chile. Mean 30-day CPUE was calculated as the mean hourly CPUE and multiplied by 720 (24 hours* 30 days). The x-axis is a square root scale. Species CPUE values are comparable within islands but not across islands because study methodology and duration differed on each island. Five breeding colonies were monitored from 2015–2020 on Isla Mocha and one breeding colony was monitored from 2019–2020 on Isla Robinson Crusoe.

On Isla Mocha, we identified 19 vertebrate species/species categories, including four introduced mammal species (Table 1, Fig 1). Pink-footed shearwaters were the most commonly detected species, with an order of magnitude greater 30-day CPUE and number of observations than the next most detected species (Table 1, Fig 1). Introduced mammals observed, in order of greatest to least CPUE, were rats, cats, dogs, and European hares (*Lepus europaeus*; Table 1, Fig 1). Native terrestrial bird species were also frequently observed, including the Mocha chucao tapaculo (*Scelorchilus rubecula mochae*), Mocha austral thrush (*Turdus falklandii mochae*), black-faced ibis (*Theristicus melanopis*), black throated huet (*Pteroptochos tarnii*), Chilean pigeon (*Patagioenas Araucana*), and Patagonian Sierra-Finch (*Phrygilus patagonicus*; Table 1, Fig 1). A variety of other native birds and mammals were observed at levels of <100 observations (Table 1, Fig 1). S2 and S3 Figs show example camera images.

**Table 1:** Detections of animals from trail cameras at five pink-footed shearwater colonies on Isla Mocha, Chile, during 2015–2020.

Latin Name	English name	Class	Native/Introduced	# Observations	30-day CPUE	30-day CPUE SD
*Ardenna creatopus*	pink-footed shearwater	bird	native	17,083	50.36	39.71
*Rattus spp*.	rat	mammal	introduced	1,110	4.80	7.79
*Scelorchilus rubecula mochae*	Mocha chucao tapaculo	bird	native (endemic)	817	4.26	4.49
*Turdus falklandii*	austral thrush	bird	native	799	4.16	3.62
*Theristicus melanopis*	black-faced ibis	bird	native	501	2.26	2.38
*Pteroptochos tarnii*	black-throated huet huet	bird	native	346	1.84	1.64
*Felis catus*	cat	mammal	introduced	247	1.27	1.29
*Patagioenas araucana*	Chilean pigeon	bird	native	268	1.07	1.75
*Phrygilus patagonicus*	Patagonian sierra-finch	bird	native	185	1.03	2.17
*Pudu puda*	pudú	mammal	unknown	83	0.36	1.32
*Canis lupus familiaris*	dog	mammal	introduced	18	0.31	1.24
*Homo sapiens*	human	mammal	NA	19	0.30	1.49
*Rodentia spp*.	rodent spp.	mammal	native	27	0.27	0.73
*Zonotrichia capensis chilensis*	rufous-collared sparrow	bird	native	28	0.18	0.50
*Cathartes aura*	black vulture	bird	native	18	0.12	0.37
*Lepus europaeus*	European hare	mammal	introduced	27	0.11	0.31
*Spinus barbatus*	black-chinned siskin	bird	native	10	0.06	0.32
*Aphrastura spinicauda bullocki*	Mocha thorn-tailed rayadito	bird	native (endemic)	13	0.06	0.15
*Eugralla paradoxa*	ochre-flanked tapaculo	bird	native	1	<0.01	0.02

Camera deployment dates are in S1 Fig. Number (#) observations is the sum of the maximum number of individuals of each species observed per hour. 30-day CPUE is the mean hourly CPUE (averaged across day and cameras) multiplied by 720 (24 hours* 30 days). Due to methodological differences between islands, CPUE and # observations metrics are specific to Isla Mocha and are not comparable to Isla Robinson Crusoe (Table 2).

### Isla Robinson Crusoe

We identified 14 vertebrate species and two non-species categories (“unknown animal” and seabird *spp*.) at PAG. Identified species included seven introduced mammals, one introduced bird, and five native birds (Table 2). European rabbits had the greatest 30-day CPUE and number of observations, followed by the “unknown animal” category and pink-footed shearwaters (Table 2, Fig 1). Austral thrushes (*Turdus falklandi*), cats, cattle, and rats, respectively, were the next most detected species (Table 2, Fig 1). Other introduced species observed were dogs, rock pigeons (*Columba livia*), house mice, and southern coati (Table 2, Fig 1). Other native species observed were Juan Fernández petrel (*Pterodroma externa*), Juan Fernández kestrel (*Falco sparverius fernandensis*), and short-eared owl (*Asio flammeus*; Table 2, Fig 1). S2 and S3 Figs show example camera images.

**Table 2:** Detections of vertebrate animals from four trail cameras at the Piedra Agujereada pink-footed shearwater colony on Isla Robinson Crusoe, Chile, during February 2019-February 2020.

Latin name	English name	Class	Native/Introduced	# Observations	30-day CPUE	30-day CPUE SD
*Oryctolagus cuniculus*	European rabbit	mammal	introduced	5,439	43.32	34.03
Unknown Animal	NA	NA	NA	2,597	20.66	29.89
*Ardenna creatopus*	pink-footed shearwater	bird	native	854	6.12	7.65
*Turdus falklandii*	austral thrush	bird	native	366	3.21	3.07
*Felis catus*	cat	mammal	introduced	263	3.17	3.42
*Bos taurus*	cow	mammal	introduced	390	2.93	3.01
*Rattus spp*.	rat	mammal	introduced	263	2.92	4.80
*Canis lupus familiaris*	dog	mammal	introduced	84	0.85	0.83
Seabird *spp*.	NA	NA	NA	36	0.47	0.68
*Columba livia*	rock pigeon	bird	introduced	38	0.43	1.04
*Homo sapiens*	human	mammal	NA	35	0.37	0.24
*Pterodroma externa*	Juan Fernández petrel	bird	native	17	0.25	0.37
*Mus musculus*	house mouse	mammal	introduced	7	0.10	0.20
*Nasua nasua*	southern coati	mammal	introduced	6	0.09	0.20
*Falco sparverius fernandensis*	Juan Fernández kestrel	Bird	native (endemic)	6	0.08	0.16
*Asio flammeus*	short-eared owl	bird	native	2	0.02	0.06

Camera deployment dates are in S1 Fig. Number (#) observations is the sum of the maximum number of individuals of each species observed per hour. 30-day CPUE is the mean hourly CPUE (averaged across day and cameras) multiplied by 720 (24 hours* 30 days). Due to methodological differences between islands, CPUE and # observations metrics are specific to Isla Robinson Crusoe and are not directly comparable to Isla Mocha (Table 1).

### Pink-footed shearwater seasonal and diel patterns

**Fig 2:**
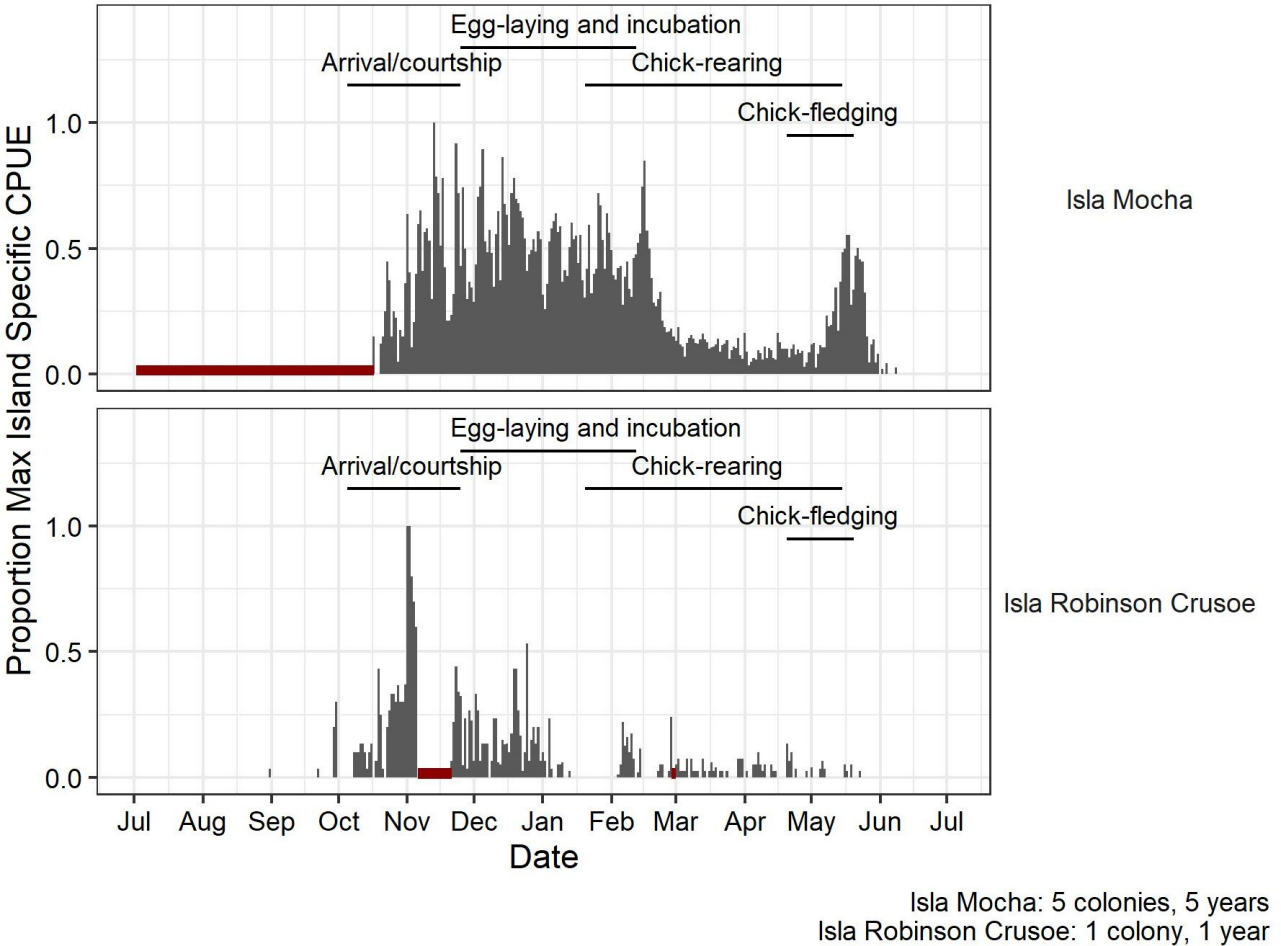
Pink-footed shearwater island-specific attendance from trail cameras on Isla Mocha and Isla Robinson Crusoe, Chile, and pink-footed shearwater breeding phenology. Cameras were deployed on Isla Mocha (n = 1–4 per colony) at five colonies with deployment times ranging from mid-October to early June from 2015–2020. Cameras (n = 4) were deployed at on colony on Isla Robinson Crusoe from February 2019-February 2020. Bar length indicates the proportion of the island-specific maximum CPUE for each day on each island. Proportion of island-specific maximum CPUE was calculated by dividing the mean hourly pink-footed shearwater CPUE by the maximum recorded hourly CPUE on each island, and averaging the hourly results for each day. This relative seasonal and diel CPUE metric is a unitless proportion and the magnitude cannot be compared across islands because of differing maximum of CPUE values, but it emphasizes relative within-species attendance patterns. Red line indicates no data. Black line indicates effort without detections on that date. Breeding stage phenology is based on long-term breeding biology studies on both islands; breeding phenology is similar on both islands [10].

**Fig 3:**
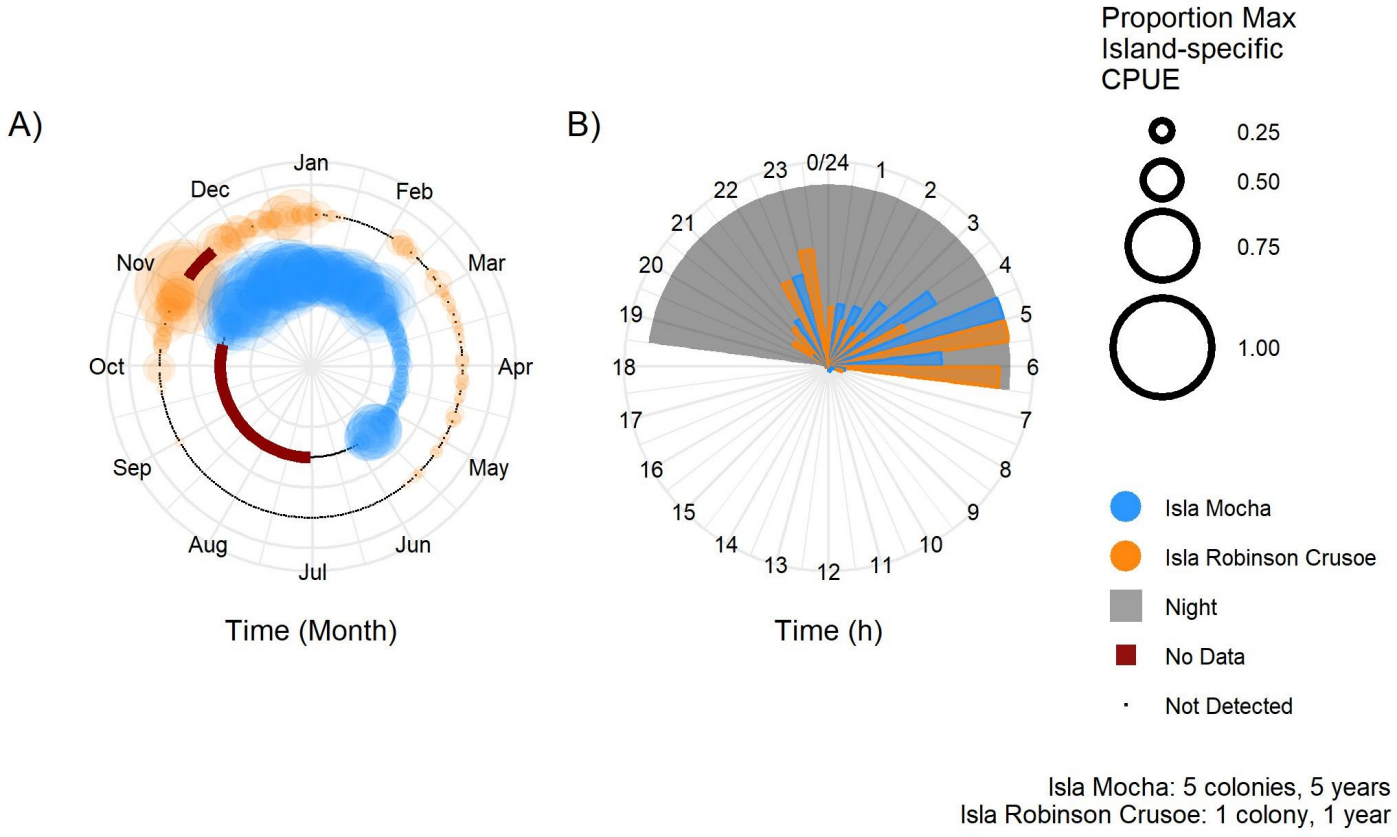
Seasonal (A) and diel (B) island-specific attendance of pink-footed shearwaters on Isla Mocha (inside circle, blue; five colonies, 2015–2020) and Isla Robinson Crusoe (outside circle, orange; one colony, 2019–2020), Chile. Proportion of island-specific maximum CPUE was calculated by dividing each species’ hourly CPUE by the maximum recorded hourly CPUE for that species on each island, and averaging the hourly results for each day for the seasonal metric (A) and for each hour of the day for the diel metric (B). This unitless relative CPUE metric can be used to compare seasonal and diel attendance across islands, but does not reflect actual magnitude or relative abundance of species because the maximum CPUEs were species- and island-specific. Circle size (A) and bar length (B) indicate the proportion of the island-specific maximum CPUE for each day (A) or hour (B) on each island. The largest point and the longest bar for each species represent the highest CPUE for that species on that island. On panel A, red line indicates no data and black line indicates effort without detections on that date.

On both islands, 30-day CPUE patterns corresponded with known pink-footed shearwater phenology stages (Figs 2 and 3A). The most evident pattern was greater 30-day CPUE in the early breeding season followed by reduced CPUE during the chick-rearing period (Figs 2 and 3A). Seasonally, the earliest detection of pink-footed shearwaters on either island was on 31 August in 2019 on IRC. On Isla Mocha, the earliest seasonal pink-footed shearwater detection in the austral spring was 16 October, 2016, which was also the earliest camera deployment date, so the first pink-footed shearwater arrival was likely earlier. CPUE was high from November through February, corresponding with the courtship, egg laying, incubation, and early chick-rearing periods (Figs 2 and 3A). CPUE decreased in late February, by which time most chicks had hatched, and stayed relatively low until an increase in May corresponding to the timing of chick fledging. In two years with camera monitoring into late June, the latest dates a shearwater was observed were 8 June, 2016 and 4 June, 2019 (Figs 2 and 3A). Shearwaters appeared with regularity on IRC starting the last week of September and were detected virtually every night from 8 October 2019 onward (Figs 2 and 3A). The 30-day CPUE peaked during November, corresponding to the courtship period, and was lower during the December and January egg-laying and incubation period (Figs 2 and 3A). The 30-day CPUE decreased again after January and was consistently low in February (late incubation period to early chick-rearing) through the end of the breeding season in May (Figs 2 and 3A). The latest detection date of a pink-footed shearwater on IRC was 23 May 2019.

On both islands, pink-footed shearwaters were active on the surface of the colony only nocturnally (Fig 3B). There were two peaks in shearwater CPUE—one during the early night (21:00–23:00), presumably as birds arrived at the colony, and a larger peak during 03:00–06:00, as birds prepared to depart (Fig 3B). The early morning peak in CPUE on IRC was from 05:00–06:00, compared to 03:00–0:400 on Isla Mocha, likely because of the later sunrise time on IRC, which is 660 km west of the South American continent but is in the same time zone as the nearshore Isla Mocha.

### Seasonal and diel patterns of introduced mammals

**Fig 4:**
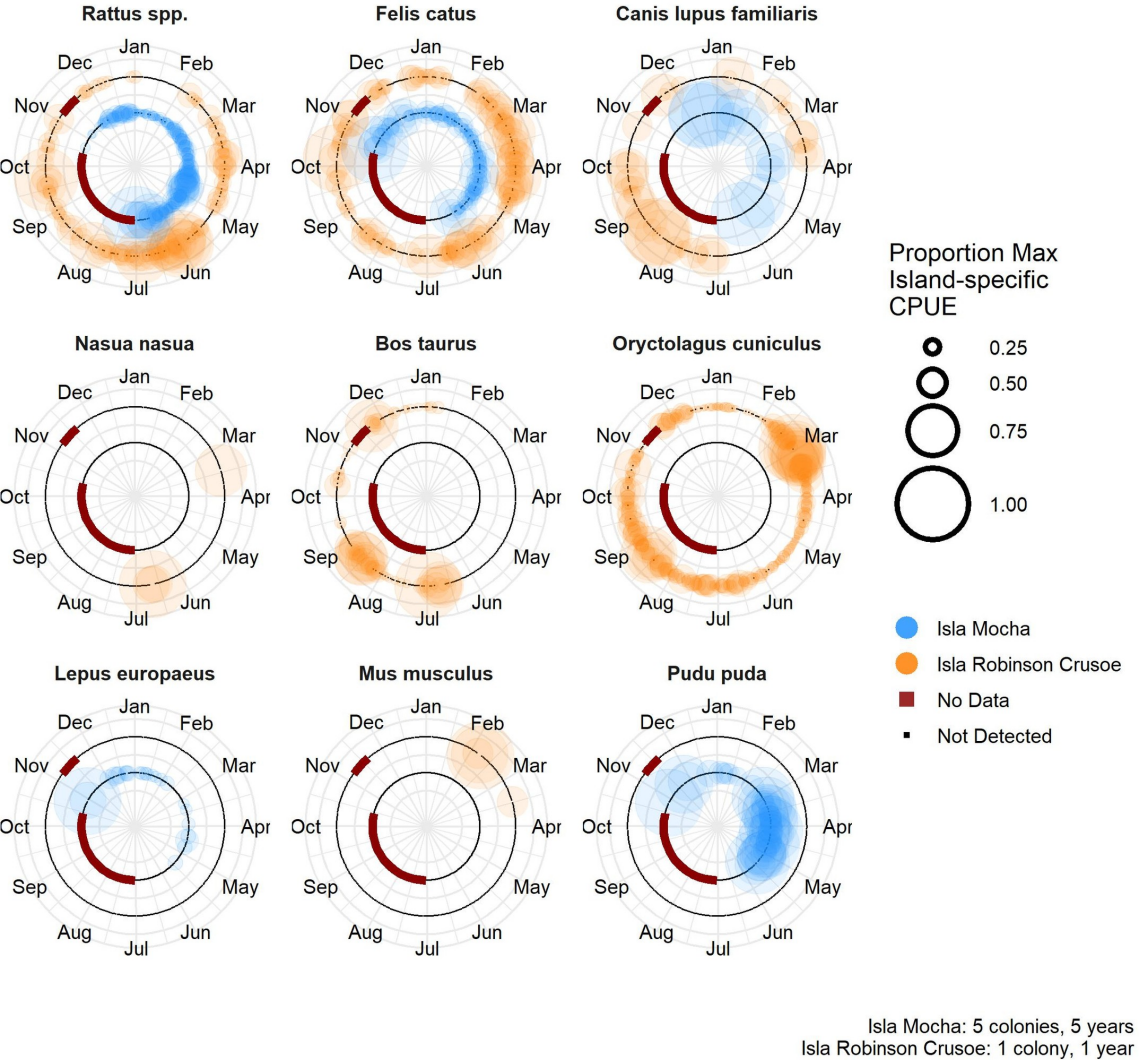
Seasonal island-specific attendance of mammals in pink-footed shearwaters colonies on Isla Mocha (inside circle, blue; five colonies, 2015–2020) and Isla Robinson Crusoe (outside circle, orange; one colony, 2019–2020), Chile. Proportion of island-specific maximum CPUE was calculated by dividing each species’ hourly CPUE by the maximum recorded hourly CPUE for that species on each island, and averaging the hourly results for each day. This unitless relative CPUE metric can be used to compare seasonal attendance across islands, but does not reflect actual magnitude or relative abundance of species because the maximum CPUEs were species- and island-specific. Circle size indicate*s* the proportion of the island-specific maximum CPUE for each day on each island. The largest point represent*s* the highest CPUE for that species on that island. Red line indicates no data. Black line indicates effort without detections on that date. Species with no colored data points were not observed on that island. All mammals shown were introduced with the exception of the pudú (Pudu pada), whose native status was uncertain.

**Fig 5:**
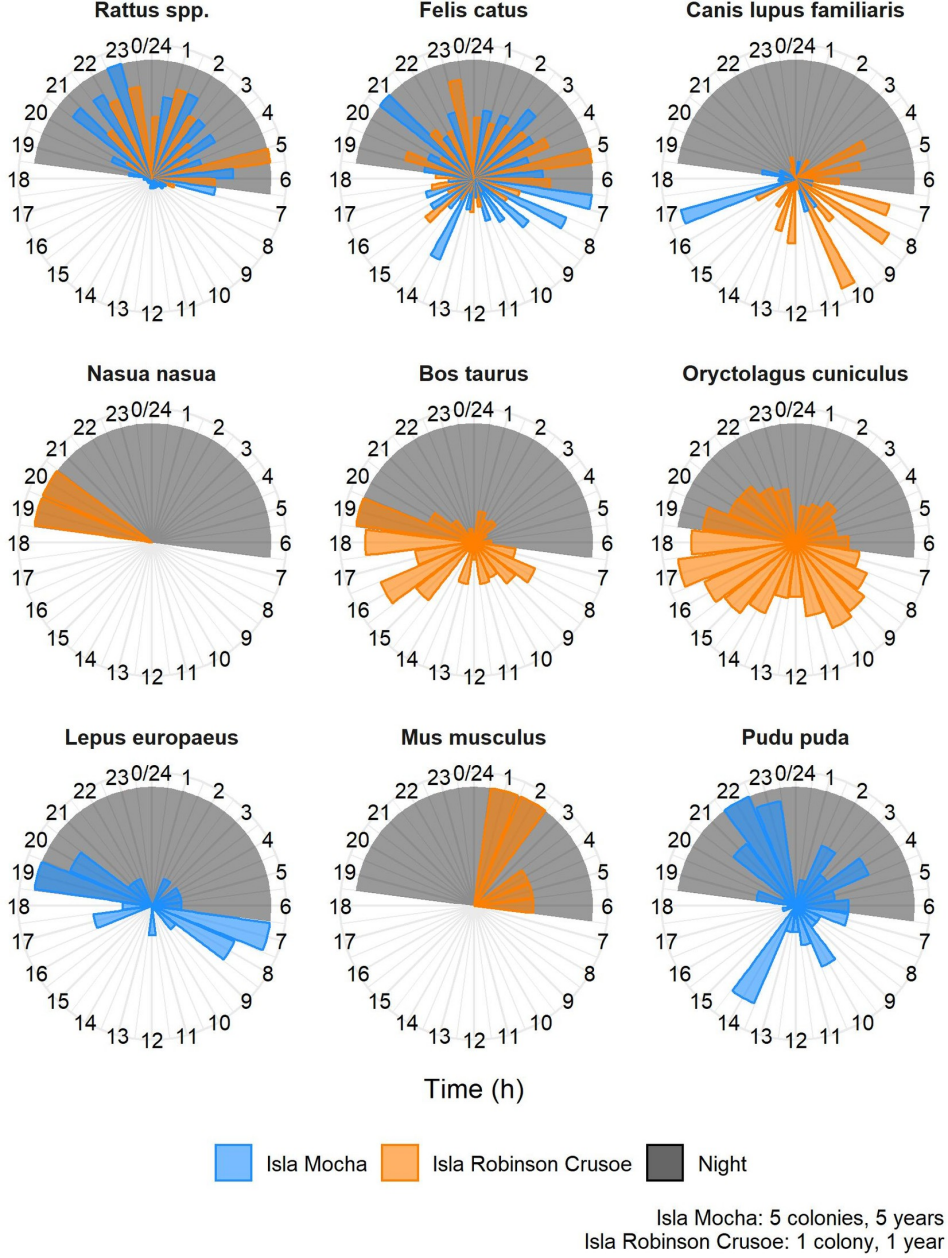
Diel island-specific attendance of mammals in pink-footed shearwaters colonies on Isla Mocha (inside circle, blue; five colonies, 2015–2020) and Isla Robinson Crusoe (outside circle, orange; one colony, 2019–2020), Chile. Proportion of island-specific maximum CPUE was calculated by dividing each species’ hourly CPUE by the maximum recorded hourly CPUE for that species on each island, and averaging the results for each hour of the day. This unitless relative CPUE metric can be used to compare diel attendance across islands, but does not reflect actual magnitude or relative abundance of species because the maximum CPUEs were species- and island-specific. Bay size indicates the proportion of the island-specific maximum CPUE for each day on each island. The longest bar represents the highest CPUE for that species on that island. All mammals shown were introduced with the exception of the pudú (Pudu pada), whose native status was uncertain.

On IRC, rats were detected in all months, with the lowest CPUE during December and January (Fig 4). Rat CPUE on IRC was lower during October-February, roughly corresponding to austral summer, than during March-September, corresponding to austral winter, the wet season on the Juan Fernández Islands (Fig 4). Rats were present during all monitoring months (October-June) on Isla Mocha, with greatest CPUE in May and June (Fig 4). Rats were detected almost exclusively during nighttime and crepuscular hours on both islands, though there were a small number of daylight observations on Isla Mocha (Fig 5).

Cats were found in pink-footed shearwater colonies on both islands year-round, with no apparent seasonal differences in relative abundance (Fig 4). Cats were active during the day and night on both colonies, with more observations at night on IRC than during the day, whereas cats on Isla Mocha were often documented during 07:00–08:00 hours (Fig 5). Dogs were sporadically observed on both islands, with no clear seasonal trends in presence, and during both night and day (Figs 4 and 5).

Introduced mammals observed only on IRC were coati, cattle, and rabbits. There were six total observations of coati, occurring in March, April, and June during the 18:30–21:30 hours (Figs 4 and 5). Cows were absent during March-June, but were regularly captured by cameras the rest of the year (Fig 4). Cows were recorded by cameras at virtually all hours of the day and night (Fig 5). Rabbits were consistently observed during all months (Fig 4), with CPUE of rabbits highest during the day and crepuscular hours; however, rabbits were recorded consistently at lower levels during all hours of the night (Fig 5). On Isla Mocha, both European hares and pudú were recorded during all monitoring months except June, and were active during both day and night (Figs 4 and 5).

### Other species

Other native species on Isla Mocha were observed consistently during all seasons (e.g. chucao tapaculo, black-throated huet huet) or were not observed frequently enough to assess seasonality (S4 Fig). There were strong diel patterns of native birds on Isla Mocha, with all bird species other than the pink-footed shearwater recorded nearly exclusively during the day (S5 Fig). On IRC, Juan Fernández petrels, which breed in the Juan Fernández Archipelago but not on IRC [35], were observed between February and June, always at night or during crepuscular hours (S4 and S5 Figs). Rock pigeons were observed only between February and June, and austral thrushes were observed at PAG in all months except February and March (S4 Fig). Similar to Isla Mocha, terrestrial bird species were observed primarily during the day at PAG (S5 Fig). Humans were observed infrequently at both islands. On IRC, all human records were researchers, whereas on Isla Mocha there were records of humans identified as “tourists” on ten separate days. In one instance on Isla Mocha, a dog was recorded approximately one minute before and after the presence of a human visitor.

### Presence of species among Isla Mocha colonies

We observed pink-footed shearwaters, cats, and rats at all of the five monitored breeding colonies on Isla Mocha. Hares and dogs were observed only in two colonies (Colonies 2 and 3), both located near trails. Pudú were observed at all colonies except Colony 1, which is bisected by a regularly used tourist hiking trail. Humans identified as “tourists” were observed only at Colony 1 and 3, both of which had trails nearby. We did not make further comparisons of relative abundance or attendance of animals in each colony on Isla Mocha because of lack of standardization of trail camera effort on that island.

## Discussion

We documented multiple species of introduced mammalian predators and herbivores within pink-footed shearwater colonies on Isla Mocha and IRC. With the exception of the European hare on Isla Mocha, the presence of the mammalian species we documented was previously described for these islands. However, our results provide the first detailed information on presence and relative abundance of introduced species within pink-footed shearwater breeding colonies, and an update and contemporary baseline for the status of introduced species on Isla Mocha and IRC. Collectively, Isla Mocha and IRC hold ≥90% of the world breeding population of pink-footed shearwaters [9]. The other approximately 10% nests on Isla Santa Clara, a smaller island adjacent to IRC in the Juan Fernández Archipelago [9], which is the only pink-footed shearwater breeding colony free of introduced vertebrates. Thus, our results advance our knowledge of an important conservation threat to at least 90% of the world population of this endangered seabird by confirming the presence in pink-footed shearwater colonies, and temporal overlap with shearwaters, of introduced mammals including cats, dogs, and rats on both islands, and rabbits, coati, and cattle on IRC. Results especially noteworthy for pink-footed shearwater conservation were that cats were continually present year-round in shearwater colonies on both islands (although several months were not monitored on Isla Mocha), that CPUE of rabbits was greater than shearwaters on IRC, and that rats were the most observed vertebrates after shearwaters on Isla Mocha.

**Fig 6:**
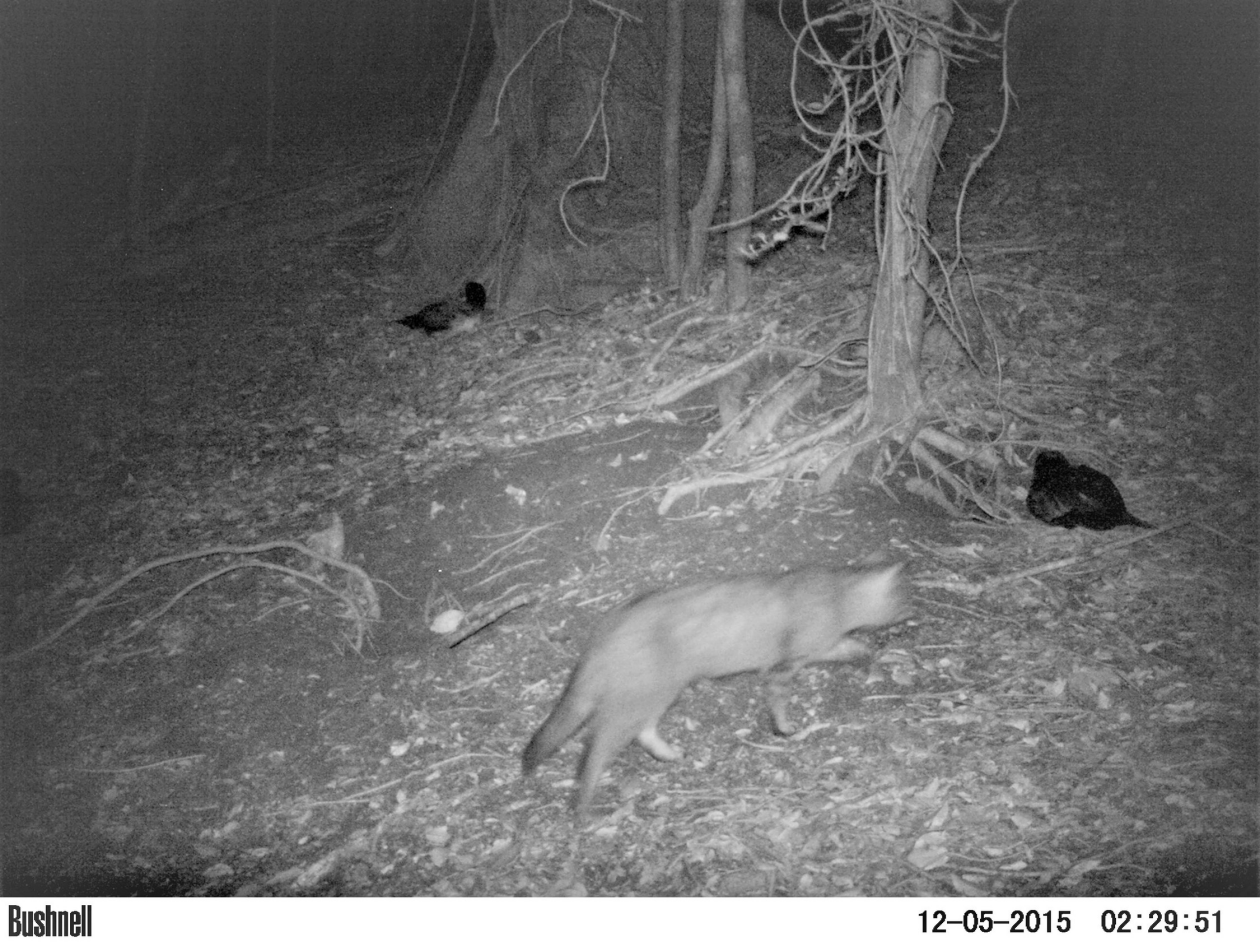
Trail camera image of a cat near two pink-footed shearwaters on Isla Mocha.

Though our study was not designed to directly assess the threats of each species to pink-footed shearwaters, there is ample evidence that introduced cats, rats, and dogs depredate burrow-nesting seabirds on other islands [36–38], and direct observations of coati killing pink-footed shearwaters on IRC (P. Hodum, P. Manríquez Angulo, G. De Rodt, pers. obs.). Trail cameras did not directly capture pink-footed shearwaters being depredated, though they recorded images of cats and dogs near pink-footed shearwaters (Fig 6), cats and rats entering burrows, and dogs digging at shearwater burrows on Isla Mocha. Pink-footed shearwaters, like many seabirds that breed on formerly predator-free islands, appear relatively, but not entirely, naïve to the risk of predation (e.g., they are easily approached and caught by hand by researchers in the breeding colony during initial captures, but flee during subsequent capture attempts; R. Carle, pers. obs.). Depredation of pink-footed shearwater adults and chicks regularly occurs on IRC, though it is typically difficult to identify the responsible predator. Whether or not these introduced predators kill pink-footed shearwaters, they likely cause indirect effects on shearwater fitness through responses such as heightened stress and increased predator avoidance behaviors [39, 40]. In other bird species, such non-lethal effects of predators have influenced reproductive behavior, nest site selection, reproductive output, and even composition of immune factors in eggs [39, 40, 41]. In a study of pink-footed shearwater colonies in the Juan Fernández Archipelago, differences in the presence of cats, rats, dogs and coati across breeding colonies did not have a measureable impact on pink-footed shearwater burrow occupancy or hatching success [18]. However, predation of pink-footed shearwaters might occur more often during the courtship and/or chick-fledging stages than during incubation. Pink-footed shearwater CPUE was greatest during the arrival/courtship stage on both islands, likely making them vulnerable to predation at this time, and chicks are vulnerable to predation when they are fledging.

The primary limitation of our study was that trail camera monitoring on Isla Mocha was not standardized. This limited our ability to draw conclusions about inter-annual trends and inter-island spatial differences in relative abundance of animals. We acknowledged this important limitation and attempted to nevertheless use this valuable long-term, multi-colony monitoring data-set from the largest pink-footed shearwater breeding colony to its full potential. However, our CPUE results for pink-footed shearwaters matched well with known breeding phenology of the species, suggesting that the metric was biologically accurate for shearwaters. Our methods provide a template for quantifying unstandardized trail camera datasets collected for conservation purposes by binning data collected with variable settings into standardized time intervals analyzed at the lowest-common-denominator resolution. Though our results must be interpreted cautiously, we show that useful information on presence and relative seasonal and diel patterns of animals can be drawn from unstandardized trail camera datasets and applied to conservation planning. We also caution that the metrics we derived from trail cameras are representative only of what the trail cameras could perceive. More conspicuous animals (e.g., cattle) were more probably more likely to trigger cameras than small and cryptic ones (e.g., mice). Other factors that could influence the seasonal or species-specific probabilities of detecting animals were seasonal changes in vegetation (especially at PAG where annual grasses and forbs grow taller in the wet season) and diurnal vs. nocturnal behavior (i.e., animals might be less visible in night photos than daytime photos). We qualitatively discuss factors likely to affect detection probabilities of various species in the interpretation of results.

The near-constant presence of cats in shearwater colonies on both islands (year-round on IRC, and during all monitored months on Isla Mocha) is a serious conservation concern for pink-footed shearwaters. Though it was previously known that cats existed on these islands, these are the first data indicating the scope of the threat of cats as a near-constant presence in pink-footed shearwater colonies. Cats are seabird predators worldwide [42, 43]. Pink-footed shearwater remains have been found in cat scat on IRC [44], and historical accounts suggest that cats, present on IRC since at least the 1600s, may have caused historic declines in shearwater abundance [26]. On both Isla Mocha and IRC, the presence of cats overlapped with the entire shearwater breeding season, indicating the likelihood of cats depredating shearwaters. Year-round camera monitoring on IRC indicated that cats were also present at similar levels in months when shearwaters were absent (we did not have data from Isla Mocha for those months), suggesting that there is sufficient non-shearwater prey at PAG, likely rabbits and rats, to support them. Not surprisingly, cats were also active at night at the same time as shearwaters, though on Isla Mocha cats were also often active during the day, which could be related to the presence of diurnal forest birds as potential prey there. Though we did not attempt to estimate absolute abundances for most animals observed in this study, we were able to use distinctive pelage colorations to identify at least five individual cats present during the year of monitoring at PAG. An important question for management of cats is whether those observed in shearwater colonies were feral or were pets or semi-domesticated individuals, because social perceptions and values of the local communities are key to successful control or mitigation of cats (e.g., cat eradication, spay and neuter programs, or other responsible pet ownership programs). That cats were present continually all year, and were recorded at all hours of the day in breeding colonies, suggests that some cats likely lived in or near the colonies. At PAG, one cat was recorded repeatedly visiting a burrow and eventually emerging with kittens, proving that at least this individual lived in the colony. However, the distance from human settlement to shearwater colonies on either island (≤1 km for Mocha colonies, 4.6 km for PAG) is within the home ranges of male cats (i.e. average linear home range 6.34 km for males, 3.83 km for females) [45], so it is possible that some cats in the breeding colonies came from towns. No cats observed appeared to be wearing collars, though domestic cats often are not collared on these islands.

Rats, present on both islands, had a particularly high CPUE on Isla Mocha, and were the second most observed vertebrate there after shearwaters. Rats negatively impact seabirds worldwide [6, 46], but direct evidence for rat depredation of pink-footed shearwaters is scarce. One author described finding eggshells chewed by rats on Isla Mocha and several chicks that were “certainly” killed by rats, but presented only descriptive evidence [47]. Notably, rat CPUE at IRC was lowest during the early pink-footed shearwater breeding season in November-February, when eggs would be vulnerable to rat predation. Rat CPUE was greater on both Isla Mocha and IRC in the wet austral winter compared with the dry austral spring/summer, suggesting that rat CPUE could be tied to availability of vegetative food sources linked with seasonal precipitation. Alternatively, rat CPUE may have been affected by seasonal differences in the visibility of rats to camera traps, for instance if vegetation growth in the wet season obscured views of rats. Given rat CPUE was greater during the wet season than the dry season, however, it seems unlikely that vegetation-related changes in rat visibility drove this pattern. Despite relatively lower CPUE of rats during the pink-footed shearwater breeding season, rats could still depredate shearwater eggs or chicks. Further study of the impacts of rats on pink-footed shearwaters is needed.

Dogs, coati, and mice are other potential predators of pink-footed shearwaters that were infrequently recorded in our study. Dogs are documented seabird predators on other islands [48, 49]. Trail cameras captured a dog digging into a pink-footed shearwater burrows on one occasion on Isla Mocha (S2C Fig). CPUE of dogs was relatively low compared with cats on both islands, and attendance patterns suggested relatively infrequent visits to colonies compared with cats. Also, dogs visited shearwater breeding colonies more often during the day than at night, perhaps indicating they were not targeting nocturnally active shearwater adults. The sporadic visitation of dogs suggested they may not have been residing in or near breeding colonies. In one instance on Isla Mocha, a dog was observed about one minute before and after a single human visitor, suggesting they were associated. Dogs are known to follow tourists up trails into the shearwater colonies on Isla Mocha (R. Carle, pers. obs.), and dogs were only found in two colonies adjacent to trails, suggesting that the trails may serve as access routes through the forest. Dogs on Isla Mocha were often recognizable to local researchers as residents’ pets (T. Varela, pers. obs.), whereas on IRC it was unclear if dogs were feral or domesticated.

Coati have been observed extracting shearwaters from burrows and killing them at the Vaquería shearwater colony on IRC (P. Hodum, P. Manríquez Angulo, G. De Rodt, pers. obs.). However, coati were detected only six times at PAG. Of these, most were in months when shearwaters were not present. These results suggest minimal overlap of coati with shearwaters at PAG, though greater study is needed on the presence of coati at other shearwater colonies on IRC. We recorded house mice only on IRC, where they were infrequently observed. It was unclear whether mouse abundance was actually low or if trail cameras were not easily triggered by mice because of their small size. House mice can have devastating impacts on seabirds, but their impact is situation-specific and depends on factors such as what other introduced vertebrates are present and the availability of plant-based food resources [50–52]. Further study is needed to quantify whether or not house mice impact pink-footed shearwaters. Also, we did not observe house mice on Isla Mocha in our study. Though there is a secondary literature report of house mice from Isla Mocha [21], upon further investigation, neither we, nor those authors could identify a primary observation source (J. Croxall, pers. comm.). Further study is needed to clarify the status of house mice on Isla Mocha, especially considering its potential to impact the island’s native rodent species.

We detected various mammalian herbivores that may have negative impacts on pink-footed shearwaters. European rabbits were the most abundant species at PAG, with a CPUE more than seven times that of pink-footed shearwaters. CPUEs were affected by each species’ behavior and visibility to trail cameras, and it is possible that the diurnal attendance of rabbits was more likely to be captured than the sometimes brief nocturnal visits of shearwaters, possibly because cameras could capture animals at a greater distance during daylight. Vegetation appeared unlikely to have influenced rabbit or shearwater visibility to cameras, as rabbit CPUE was fairly consistent during all seasons except for a brief, unexplained increase in February-early April, and the relatively low shearwater CPUE from January through May preceded vegetation growth during the wet season (beginning in April to May). Despite potential differences in the ability of cameras to perceive rabbits and shearwaters, the magnitude of the difference in CPUE between the two species suggests than rabbits likely outnumber shearwaters at PAG. This, coupled with rabbits’ year-round presence at PAG, suggests that competition from rabbits for burrow space is a serious conservation concern for pink-footed shearwaters. In another study on the Juan Fernández Archipelago, presence of rabbits in shearwater colonies had a negative impact on pink-footed shearwater burrow occupancy, and burrow occupancy of pink-footed shearwaters increased on Isla Santa Clara, another island in the Juan Fernández Archipelago, after rabbit eradication [18]. Burrow occupancy of seabirds has increased after eradications of rabbits on other islands [53], likely related to rabbits outcompeting shearwaters for burrows. Though planned eradication of rabbits inside the upgraded fence at PAG should eliminate rabbit competition at that colony, rabbit competition likely threatens pink-footed shearwaters at other IRC colonies. Rabbits are not present on Isla Mocha, but given the lack of biosecurity on Isla Mocha, they easily could be introduced; in fact, residents have kept domesticated rabbits on the island in recent years though they have yet to escape captivity (R. Carle, pers. obs.)

Cattle were regularly present at PAG from June through mid-January 2019. Cattle are also present in low-lying areas of Isla Mocha but were not observed in high-elevation shearwater colonies. Cattle on IRC are managed and are grazed in the area around PAG, where they have negatively impacted shearwaters through burrow trampling and vegetation modification [32]. Trail cameras at PAG were installed inside a cattle exclusion fence constructed in 2011 to protect shearwater burrows, but it was that by 2019 cattle were breaching this fence because it was in need of maintenance. Indeed, during our study period cattle may have sought out the inside of the fence for better grazing conditions because of increased vegetation cover due to previous cattle exclusion, compared with heavily grazed areas outside the fence [32]. The fence around PAG was upgraded in 2020 (post-study) to exclude cattle, cats, dogs, coati, and rabbits, thereby eliminating new cattle impacts in the majority of the colony. Cattle were not recorded from February through May, likely because grazing conditions were less attractive there during the dry season, or because they were seasonally moved by ranchers from the area.

The only mammals on Isla Mocha that were not present on IRC were the European hare, the pudú, and native rodents. Ours is the first study documenting the presence of European hares on Isla Mocha. The European hare is considered a serious pest with negative effects on native ecosystems in continental South America [54, 55]. Hares rarely burrow [56], and thus would not be expected to compete for burrow space with shearwaters. Potential negative impacts on the native forest from hare herbivory should be considered, though during our study period, hares were infrequent in the higher elevation forests. Similar to dogs, hares were only observed in two breeding colonies on Isla Mocha, both adjacent to hiking trails, suggesting that trails may serve as an access into the forest for this species. Like hares, pudú were infrequently observed. This small deer seems unlikely to structurally damage pink-footed shearwater burrows. Herbivory from pudú may impact the native forest but they were infrequently observed, suggesting their population may be small and/or that they prefer areas we did not monitor. The only breeding colony on Isla Mocha where pudú were not observed was Colony 1, which is bisected by a popular hiking trail, suggesting that this species may avoid areas frequented by people. A recent review indicated uncertainty about whether the pudú was extant on Isla Mocha [57]; our results confirm it is extant, though it is unclear if this was due to recent reintroduction or not. Native rodents were likewise infrequently captured by trail cameras on Isla Mocha. Whether the low CPUE of native rodents was due to their small size (most are mouse-sized), cryptic behavior, habitat preferences, or actual low abundances on the island was unclear. Little is known about the native rodents of Isla Mocha and their ecology needs further study [24]. The interactions between pink-footed shearwaters and native rodents are unknown, though presumably shearwaters have long survived with the presence of native rodents on Isla Mocha. Native rodents were observed far less frequently than non-native rats.

### Seasonal and diel patterns of pink-footed shearwaters

Trail camera monitoring allowed us to assess the seasonal and diel attendance patterns of pink-footed shearwaters, which had previously been reported only generally [10]. Seasonal changes in the magnitude of shearwater CPUE from trail cameras matched well against known pink-footed shearwater phenology, suggesting that trail camera monitoring captured biologically relevant patterns of shearwater behavior. Though it is possible that factors other than reproductive phenology influenced shearwater CPUE, such as moon phase or vegetation growth in front of cameras, the similarity in CPUE patterns between islands, and its similarity to attendance patterns of other shearwaters, suggests reproductive behavior was an important factor. On both islands, Pink-footed shearwaters arrived *en masse* on both islands in early October, and shearwater CPUE was greatest during the pre-laying and incubation periods, which was similar to patterns observed in short-tailed shearwaters (*Ardenna tenuirostris*) [58] and Cory’s shearwaters (*Calonectris borealis*) [59]. This could be related to a greater presence of non-breeding adults during the early breeding period, as has been observed in other seabird species [58, 60]. On Isla Mocha, there was an increase in shearwater CPUE during May, probably related to a pulse of fledging chicks emerging from burrows. It is unclear why a similar pulse of fledging chicks was not observed in April or May at PAG, but we speculate that this could be related to the larger population of pink-footed shearwaters on Isla Mocha resulting in more fledgling chicks being visible, or possibly due growth of annual grasses at PAG obscuring fledging chicks from view of cameras. In other shearwaters, attendance at the colony and time spent outside the burrow was related to individual breeding status and sex, resulting in different exposures to cat predation [61]. We did not investigate shearwater attendance at the demographic level or predation rates, but these would be useful topics for further research on pink-footed shearwaters. Finally, the absence of shearwaters on IRC from July through September was expected, but its confirmation is useful for timing control or eradication of introduced species around periods when shearwaters are absent. We did not monitor year-round on Isla Mocha but similar confirmation of the absence of shearwaters would be useful as there have been local records of grounded pink-footed shearwater fledglings during months when shearwaters are believed to be absent (T. Varela, pers. obs.)

The strictly nocturnal behavior of pink-footed shearwaters at the breeding colony was consistent with results from a previous GPS-tracking study [12], and was similar to other shearwater and petrel species [62, 63]. Observations of shearwaters peaked during the early night and again during the hours before dawn. The nocturnal attendance of pink-footed shearwaters may be an adaptation to avoid native avian predators, however cats and coati, both likely predators of adult shearwaters, were also largely nocturnal or crepuscular. Yelkouan shearwaters (*Puffinus yelkouan*) showed peaks in colony attendance during the three hours after dusk and the two before dawn, consistent with the pink-footed shearwater attendance patterns we observed [60]. We did not investigate relationships of pink-footed shearwater diel attendance patterns with moon phase, which influenced attendance patterns in other shearwater species [63–65], though our dataset could provide more detailed information on this topic.

### Conclusions

Camera trap data proved useful for broadly describing the assemblages and patterns of native and introduced vertebrates within the breeding colonies of pink-footed shearwaters. Our results also highlight broader conservation issues on Isla Mocha and IRC, with introduced species having the potential to impact endemic and endangered plant and animal species. For example, on Isla Mocha, cats and rats may threaten multiple endemic bird sub-species [23], as well as the Pacific degu (*Octodon pacifica*) and Isla Mocha ground frog (*Eupsophus insularis*), both of which are critically endangered [66, 67]. Likewise, cats and rats and mice may threaten and the critically endangered endemic Juan Fernández Firecrown (*Sephanoides fernandensis*) hummingbird on IRC [68]. Cattle and rabbit herbivory continues to threaten endangered plant species on IRC, which has one of the highest rates of island plant endemism worldwide [31]. Our results underscore the need for rigorous biosecurity protocols for Isla Mocha and IRC to prevent further species introductions. Conservation gains can be made for the pink-footed shearwater and the unique insular ecosystems of Isla Mocha and IRC through eradication of introduced mammals from these islands, and/or from exclusion of introduced mammals from pink-footed shearwater colonies.

## Supporting information

S1 Abstract Resumen en Español. (PDF)

S1 Table Primary and secondary literature records for introduced and native mammals on Isla Mocha and Isla Robinson Crusoe (IRC), Chile. Full citations are listed in the main text of the manuscript with the exception of the articles with full citations listed below the table.
(PDF)

S2 Table Number of burrows and density of burrows in randomly selected 5 m radius containing burrows plots on Isla Mocha from a separate study. In that study, 295 points were randomly generated across Isla Mocha in areas considered suitable habitat for pink-footed shearwater breeding based on elevations and slopes. The points that had burrows present are shown.
(PDF)

S1 Fig Trail camera deployments on Isla Mocha and Isla Robinson Crusoe, with number of cameras per colony over time. PAG is the Piedra Agujereada colony on Isla Robinson Crusoe. Each “cuadrante” on Isla Mocha is a separate breeding colony.
(TIF)

S2 Fig Example trail camera images of select mammal species in pink-footed shearwater (*Ardenna creatopus*) breeding colonies on Isla Mocha and Isla Robinson Crusoe (IRC), Chile. (PDF)

S3 Fig Example trail camera images of select native bird species in pink-footed shearwater (*Ardenna creatopus*) breeding colonies on Isla Mocha and Isla Robinson Crusoe (IRC), Chile. (PDF)

S4 Fig Seasonal island-specific attendance of native bird species in pink-footed shearwater colonies on Isla Mocha (inside circle, blue; five colonies, 2015–2020) and Isla Robinson Crusoe (outside circle, orange; one colony, 2019–2020), Chile. Proportion of island-specific maximum CPUE was calculated by dividing each species’ hourly CPUE by the maximum recorded hourly CPUE for that species on each island, and averaging the hourly results for each day. This unitless relative CPUE metric can be used to compare seasonal attendance across islands, but does not reflect actual magnitude or relative abundance of species because the maximum CPUEs were species- and island-specific. Circle size indicate*s* the proportion of the island-specific maximum CPUE for each day on each island. The largest point represent*s* the highest CPUE for that species on that island. Red line indicates no data. Black line indicates effort without detections on that date. Species with no colored data points were not observed on that island.
(TIF)

S5 Fig Diel island-specific attendance of native bird species in pink-footed shearwater colonies on Isla Mocha (inside circle, blue; five colonies, 2015–2020) and Isla Robinson Crusoe (outside circle, orange; one colony, 2019–2020), Chile. Proportion of island-specific maximum CPUE was calculated by dividing each species’ hourly CPUE by the maximum recorded hourly CPUE for that species on each island, and averaging the results for each hour of the day. This unitless relative CPUE metric can be used to compare diel attendance across islands, but does not reflect actual magnitude or relative abundance of species because the maximum CPUEs were species- and island-specific. Bay size indicates the proportion of the island-specific maximum CPUE for each day on each island. The longest bar represents the highest CPUE for that species on that island.
(TIF)

S1 Data Raw data from trail camera images from Isla Mocha. (CSV)

S2 Data Raw data from trail camera images from Piedra Agujereada, Isla Robinson Crusoe. (CSV)

S3 Data Data documentation for data files from Isla Mocha and Isla Robinson Crusoe. (XLSX)
