# Supplementary material for: Introduced and native vertebrates in pink-footed shearwater (Ardenna creatopus) breeding colonies in Chile
Source: PLoS One. 2021 Jul 29;16(7):e0254416. doi: 10.1371/journal.pone.0254416 (PMC8321096; doi:10.1371/journal.pone.0254416)
Supplement: S1 Abstract — (PDF) [file pone.0254416.s001.pdf]

Resumen en Español

La planificación para la conservación de la biodiversidad requiere información precisa y actualizada sobre el estado de las especies y sus amenazas. Aunque los mamíferos introducidos son la mayor amenaza para las aves marinas a nivel mundial, faltan datos sobre especies introducidas presentes en muchas islas donde nidifican aves marinas. Para orientar la planificación de la conservación, utilizamos cámaras trampas que documentaron la presencia, abundancia relativa y visitación estacional y diaria de vertebrados introducidos y nativos dentro de las colonias reproductivas de fardela blanca (*Ardenna creatopus*) en Isla Mocha (cinco colonias, 2015-2020) e Isla Robinson Crusoe en el Archipiélago Juan Fernández (una colonia, 2019-2020), Chile. Las especies más comúnmente detectadas en Isla Mocha fueron la fardela blanca y ratas introducidas (*Rattus* spp.), y el conejo europeo (*Oryctolagus cuniculus*) y la fardela blanca en Isla Robinson Crusoe. Los mamíferos introducidos más detectados, en orden de mayor captura por unidad de esfuerzo, fueron ratas, gatos (*Felis catus*), perros (*Canis lupus familiaris*) y liebres europeas (*Lepus europaeus*) en Isla Mocha y conejos, gatos, ganado bovino (*Bos taurus*), ratas, perros, ratones (*Mus musculus*) y coatíes (*Nasua nasua*) en la Isla Robinson Crusoe. Los resultados más relevantes para la conservación de la fardela blanca mostraron la presencia de gatos durante todos los meses de monitoreo en las colonias reproductivas en ambas islas, mayor captura por unidad de esfuerzo de conejos que de fardelas en la Isla Robinson Crusoe, y la gran presencia de ratas en Isla Mocha siendo los vertebrados más registrados después de las fardelas. Las fardelas blancas estuvieron presentes regularmente en ambas islas desde octubre hasta mayo. La presencia y captura relativa por unidad de esfuerzo de fardela blanca coincide cualitativamente con la fenología reproductiva conocida para la especie. La presencia regular y la superposición temporal de fardelas con gatos, ratas, conejos y ganado bovino dentro de las colonias de fardela, junto con la presencia irregular de perros, coatís, liebres y ratones, indican una amenaza seria para la conservación de la fardela blanca y otras especies nativas. Finalmente, nuestro estudio proporciona un modelo ampliamente aplicable para el análisis de datos recopilados por varios años a través de cámaras trampas con configuraciones no estandarizadas.
