# Supplementary material for: Introduced and native vertebrates in pink-footed shearwater (Ardenna creatopus) breeding colonies in Chile
Source: PLoS One. 2021 Jul 29;16(7):e0254416. doi: 10.1371/journal.pone.0254416 (PMC8321096; doi:10.1371/journal.pone.0254416)
Supplement: S1 Table — Full citations are listed in the main text of the manuscript with the exception of the articles with full citations listed below the table. (PDF) [file pone.0254416.s002.pdf]

S1 Table. Primary and secondary literature records for introduced and native mammals on Isla Mocha and Isla Robinson Crusoe (IRC), Chile. Full citations are listed in the main text of the manuscript with the exception of the articles listed below the table.

Species	Status	Island	Published primary observation	Published secondary observation
Cat<br>*Felis catus*	Introduced – feral & domestic	IRC	Mann Wilke 1975, Hahn et al. 2002	Croxall et al. 2012, Garcia-Díaz et al. 2020, Bourne et al. 1992, Wester 1991
Dog<br>*Canis lupus familiaris*	Introduced – feral & domestic	IRC	Mann Wilke 1975, Hahn et al. 2002	Croxall et al. 2012, Garcia-Díaz et al. 2020, Wester 1991
Southern coati<br>*Nasua nasua*	Introduced	IRC	Mann Wilke 1975, Hahn et al. 2002	Croxall et al. 2012, Garcia-Díaz et al. 2020, Bourne et al. 1992, Wester 1991
Goat<br>*Capra hircus*	Introduced	IRC	Mann Wilke 1975, Hahn et al. 2002	Bourne et al. 1992, Wester 1991
European Rabbit<br>*Oryctolagus cuniculus*	Introduced	IRC	Mann Wilke 1975, Hahn et al. 2002	Croxall et al. 2012, Garcia-Díaz et al. 2020, Bourne et al. 1992, Wester 1991
Black rat<br>*Rattus rattus*	Introduced	IRC	Mann Wilke 1975	Croxall et al. 2012, Garcia-Díaz et al. 2020; Bourne et al. 1992, Hahn et al. 2002, Wester 1991

Norway rat<br><br>*Rattus norvegicus*	Introduced	IRC	Mann Wilke 1975	Croxall et al. 2012, Garcia-Díaz et al. 2020, Bourne et al. 1992, Hahn et al. 2002, Wester 1991
Rat spp.<br><br>*Rattus* spp.	Introduced	IRC	Hahn et al. 2002	
House mouse<br><br>*Mus musculus*	Introduced	IRC	Mann Wilke 1975	Croxall et al. 2012, Garcia-Díaz et al. 2020, Hahn et al. 2002, Bourne et al. 1992
Cattle<br><br>*Bos taurus*	Introduced - managed	IRC	Hahn et al. 2002, Mann Wilke 1975	Croxall et al. 2012, Garcia-Díaz et al. 2020, Bourne et al. 1992, Carle et al. 2016
Horse, donkey, mule,<br><br>*Equus caballus, E. asinus, E. asinus x caballus*	Introduced - managed	IRC	Hahn et al. 2002, Mann Wilke 1975	Wester 1991
Cat<br><br>*Felis catus*	Introduced –feral & domestic	Isla Mocha	Pefaur & Yañez 1980	Croxall et al. 2016, Hahn et al. 2016
Dog	Introduced	Isla Mocha		Hahn et al. 2016, Guicking et al. 1999

Canis lupus familiaris	–feral & domestic			
Black rat<br>*Rattus rattus*	Introduced	Isla Mocha	Pefaur & Yañez 1980	Hutterer 1994
Norway rat<br>*Rattus norvegicus*	Introduced	Isla Mocha	Pefaur & Yañez 1980	Croxall et al. 2012
Rat spp.<br>*Rattus* spp.	Introduced	Isla Mocha		Hahn et al. 2016
House Mouse<br>*Mus musculus*	Introduced	Isla Mocha		Croxall et al. 2016
Pudú<br>*Pudu pada*	Unknown	Isla Mocha	Pefaur & Yañez 1980	MacNamara & Eldridge 1987
Cattle<br>*Bos taurus*	Introduced-managed	Isla Mocha		Bahlburg & Spiske 2015
Horse, mule, donkey<br>*Equus caballus, E. asinus, E. asinus x caballus*	Introduced-managed	Isla Mocha		CONAF 1998 (unpublished)

European Hare *Lepus europaeus*	Introduced	Isla Mocha	No record before this study	
Olive grass mouse *Abrothrix olivacea* (Formerly *Akodon olivaceus*)	Native	Isla Mocha	Osgood 1943	Hutterer 1994
Long-haired grass mouse *Abrothrix longipilis* (formerly *Akodon longipilis*)	Native	Isla Mocha	Osgood 1943	Hutterer 1994
Long-tailed colilargo *Oryzomys longicaudatus*	Native	Isla Mocha	Osgood 1943	Hutterer 1994
Long-clawed mole mouse *Gioxus valdivianus* (formerly	Native	Isla Mocha	Osgood 1943	Hutterer 1994

Notiomys valdivianus)			
Pacific degu *Octodon pacificus*	Native (endemic)	Isla Mocha	Hutterer 1994

CONAF. Plan de manejo de Reserve Nacional Isla Mocha. Documento de manejo 277. Región VIII, Chile. 1998.

MacNamara, M, Eldrige, W. Behavior and reproduction in captive pudu (*Pudu puda*) and red brocket (*Mazama americana*), a descriptive and comparative analysis. In Biology and management of the Cervidae (CM Wemmer, ed) Smithsonian Institution Press, Washington, D.C. 1987.

Osgood WH. The mammals of Chile. Field Museum of Natural History; 1943
