# Supplementary material for: Introduced and native vertebrates in pink-footed shearwater (Ardenna creatopus) breeding colonies in Chile
Source: PLoS One. 2021 Jul 29;16(7):e0254416. doi: 10.1371/journal.pone.0254416 (PMC8321096; doi:10.1371/journal.pone.0254416)
Supplement: S2 Table — In that study, 295 points were randomly generated across Isla Mocha in areas considered suitable habitat for pink-footed shearwater breeding based on elevations and slopes. The points that had burrows present are shown. (PDF) [file pone.0254416.s003.pdf]

S2 Table. Number of burrows and density of burrows in randomly selected 5 m radius containing burrows plots on Isla Mocha from a separate, unpublished study by the same authors. In that study, 295 points were randomly generated across Isla Mocha in areas considered suitable habitat for pink-footed shearwater breeding based on elevations and slopes. The points that had burrows present are shown.

Point Name	Number of burrows	Burrow per m^2
A206	1	0.01
A084	4	0.05
A080	2	0.03
B070	1	0.01
B049	2	0.03
A132	9	0.11
M44	1	0.01
M48	1	0.01
B040	3	0.04
A048	5	0.06
B005	2	0.03
A015	2	0.03
A109	1	0.01
A028	1	0.01
M25	8	0.10
A114	3	0.04
M26	3	0.04
M17	3	0.04
A075	1	0.01
M23	5	0.06
A126	4	0.05
A093	6	0.08
B067	4	0.05
A120	1	0.01
