# Supplementary figures and images for: Introduced and native vertebrates in pink-footed shearwater (Ardenna creatopus) breeding colonies in Chile
Source: PLoS One. 2021 Jul 29;16(7):e0254416. doi: 10.1371/journal.pone.0254416 (PMC8321096; doi:10.1371/journal.pone.0254416)

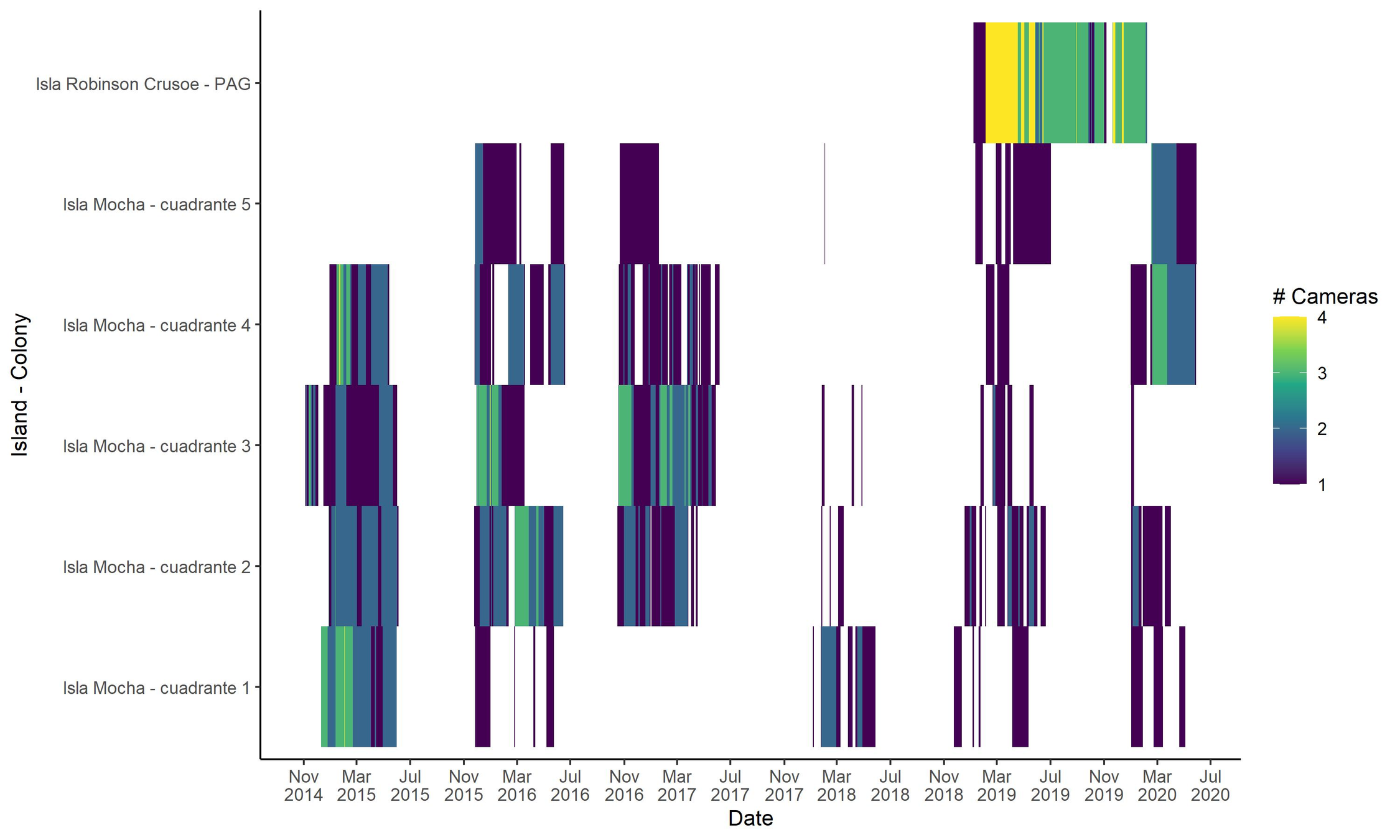

Supplement: S1 Fig — PAG is the Piedra Agujereada colony on Isla Robinson Crusoe. Each “cuadrante” on Isla Mocha is a separate breeding colony. (TIF) [file pone.0254416.s004.tif]

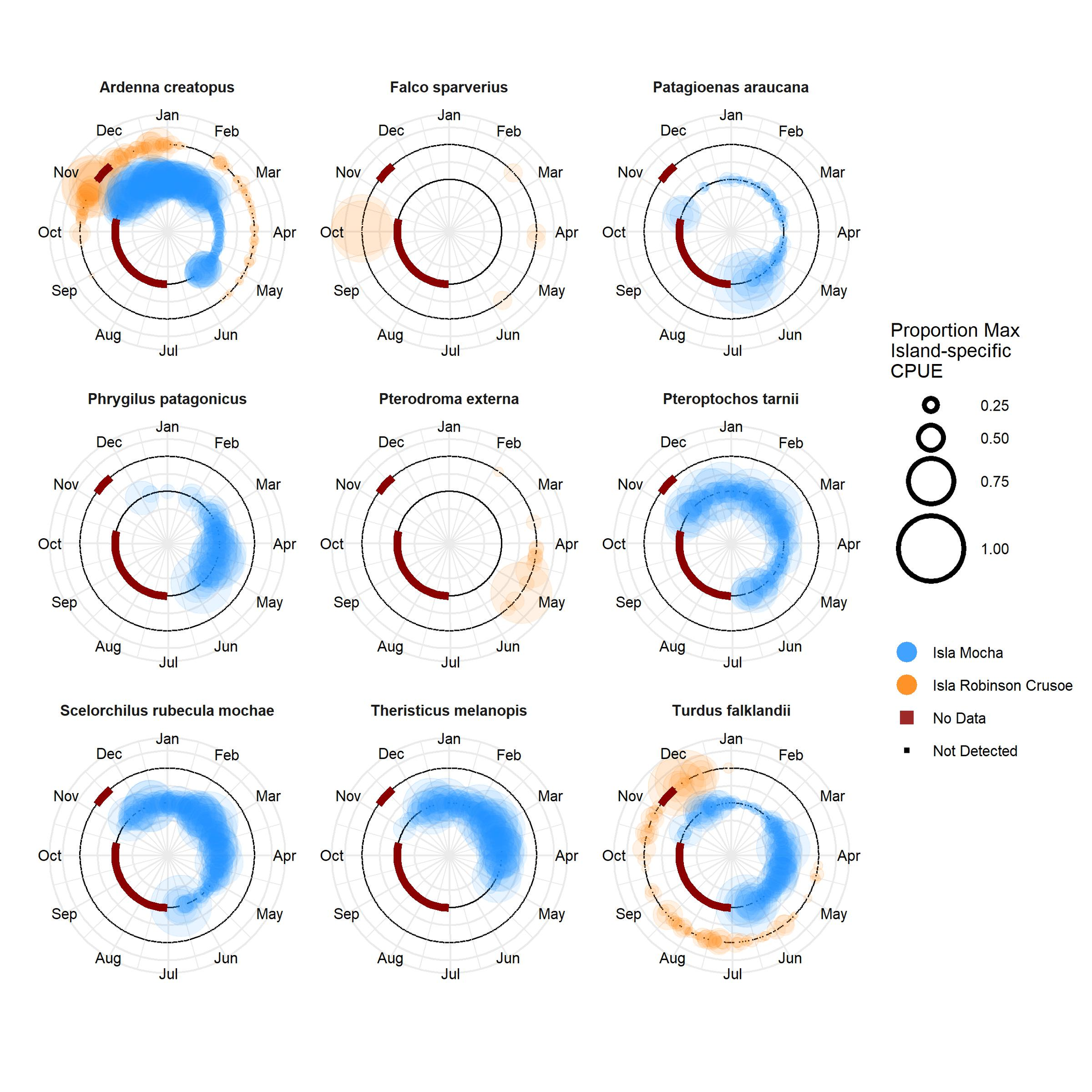

Supplement: S4 Fig — Proportion of island-specific maximum CPUE was calculated by dividing each species’ hourly CPUE by the maximum recorded hourly CPUE for that species on each island, and averaging the hourly results for each day. This unitless relative CPUE metric can be used to compare seasonal attendance across islands, but does not reflect actual magnitude or relative abundance of species because the maximum CPUEs were species- and island-specific. Circle size indicates the proportion of the island-specific maximum CPUE for each day on each island. The largest point represents the highest CPUE for that species on that island. Red line indicates no data. Black line indicates effort without detections on that date. Species with no colored data points were not observed on that island. (TIF) [file pone.0254416.s007.tif]

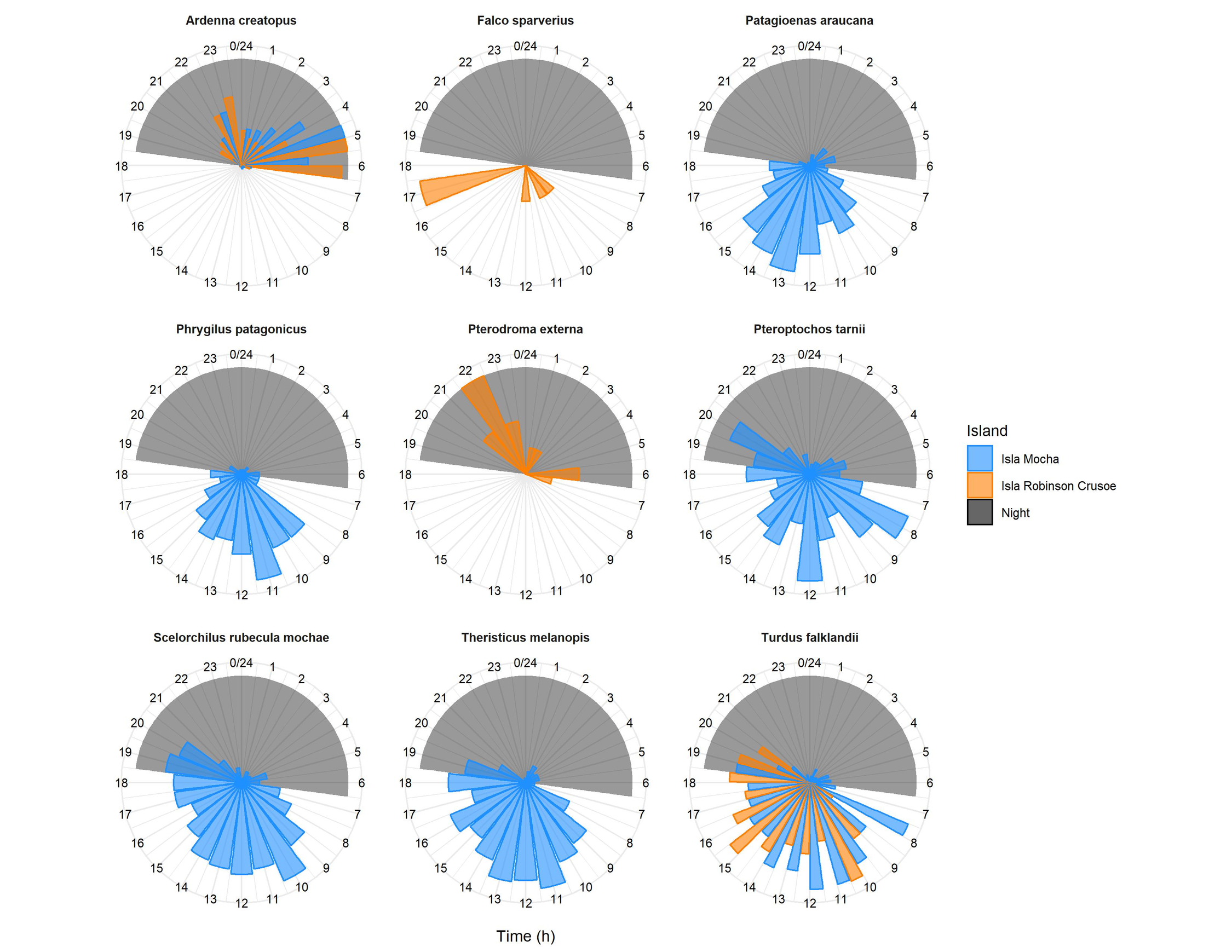

Supplement: S5 Fig — Proportion of island-specific maximum CPUE was calculated by dividing each species’ hourly CPUE by the maximum recorded hourly CPUE for that species on each island, and averaging the results for each hour of the day. This unitless relative CPUE metric can be used to compare diel attendance across islands, but does not reflect actual magnitude or relative abundance of species because the maximum CPUEs were species- and island-specific. Bay size indicates the proportion of the island-specific maximum CPUE for each day on each island. The longest bar represents the highest CPUE for that species on that island. (TIF) [file pone.0254416.s008.tif]
