# Supplementary material for: Introduced and native vertebrates in pink-footed shearwater (Ardenna creatopus) breeding colonies in Chile
Source: PLoS One. 2021 Jul 29;16(7):e0254416. doi: 10.1371/journal.pone.0254416 (PMC8321096; doi:10.1371/journal.pone.0254416)

**S2 Fig.** Example trail camera images of select mammal species in pink-footed shearwater (*Ardenna creatopus*) breeding colonies on Isla Mocha and Isla Robinson Crusoe (IRC), Chile.

A. Rat (*Rattus* spp.; bottom left of image) on Isla Mocha.

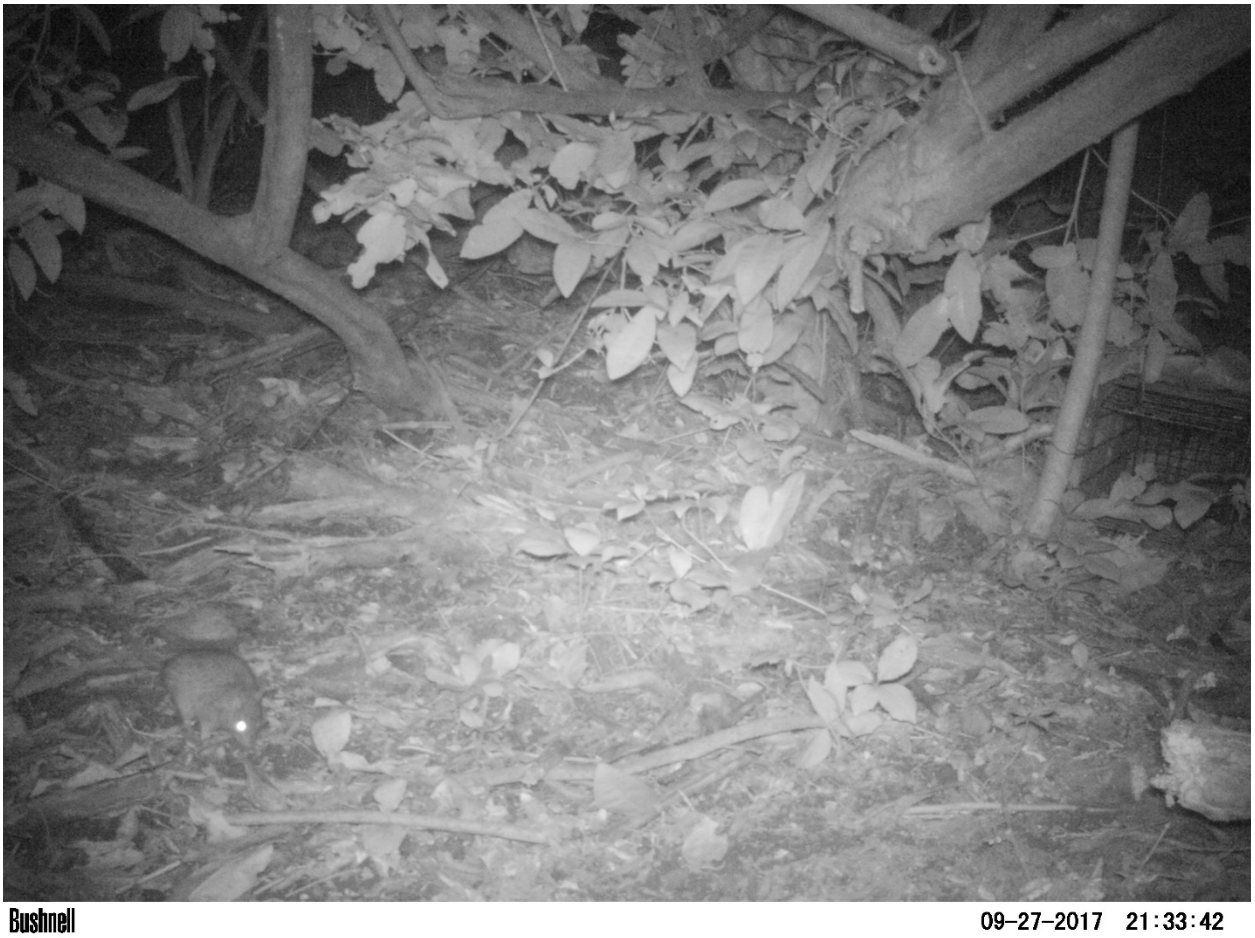

B. Cat (*Felis catus*) on Isla Mocha.

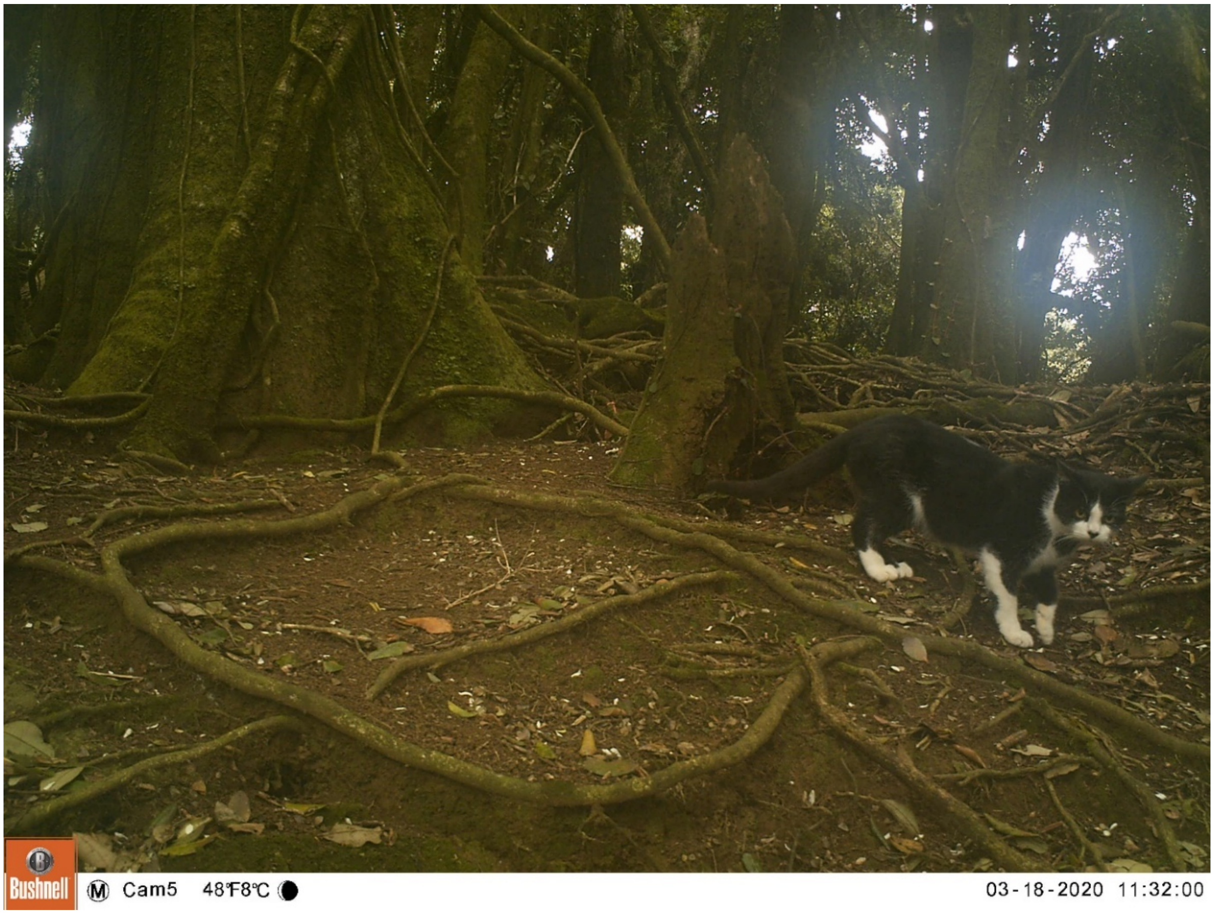

C. Dog (*Canis lupus familiaris*; bottom right at base of tree) digging into a pink-footed shearwater burrow on Isla Mocha.

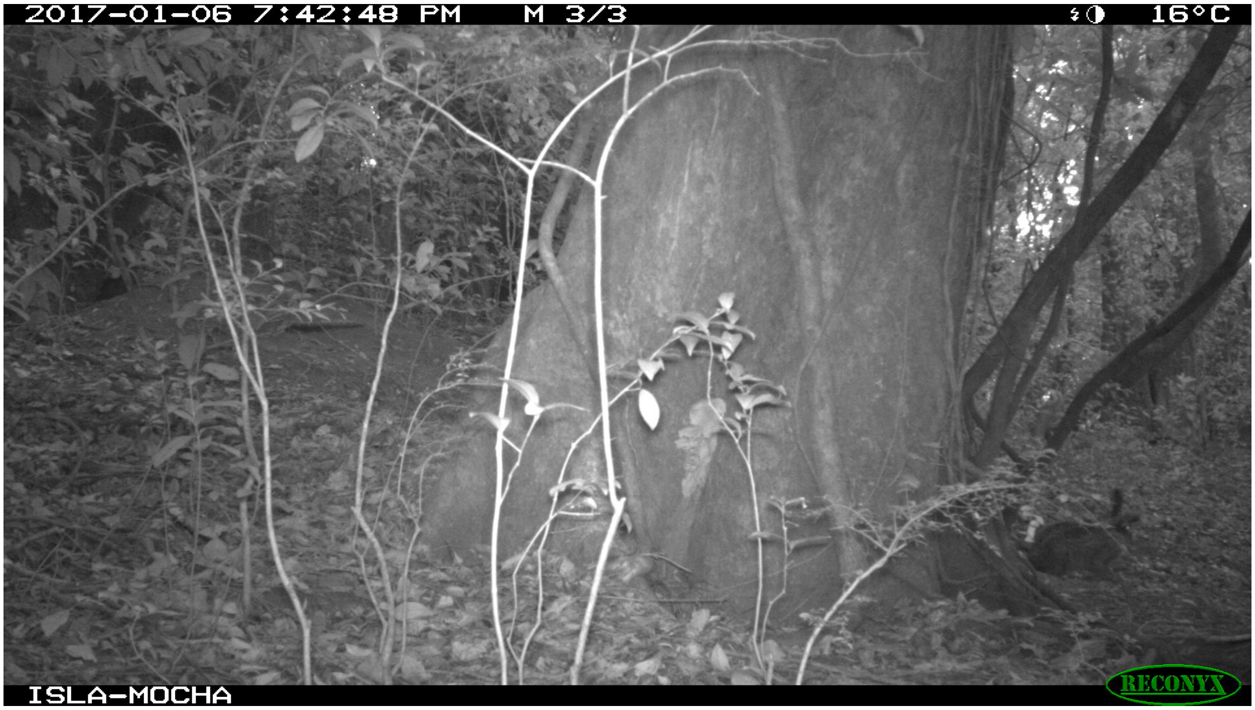


D. Pudú (*Pudu pada*) on Isla Mocha.

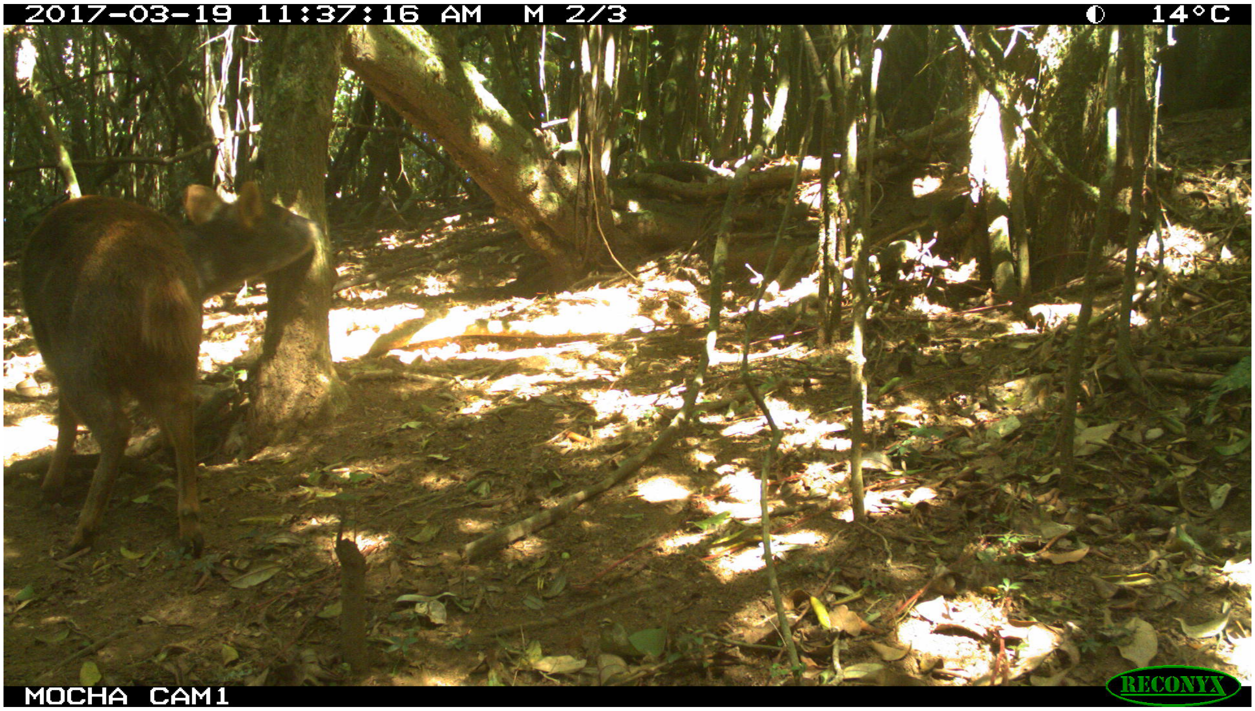

E. European rabbit (*Oryctolagus cuniculus*) at Piedra Agujereada on IRC.

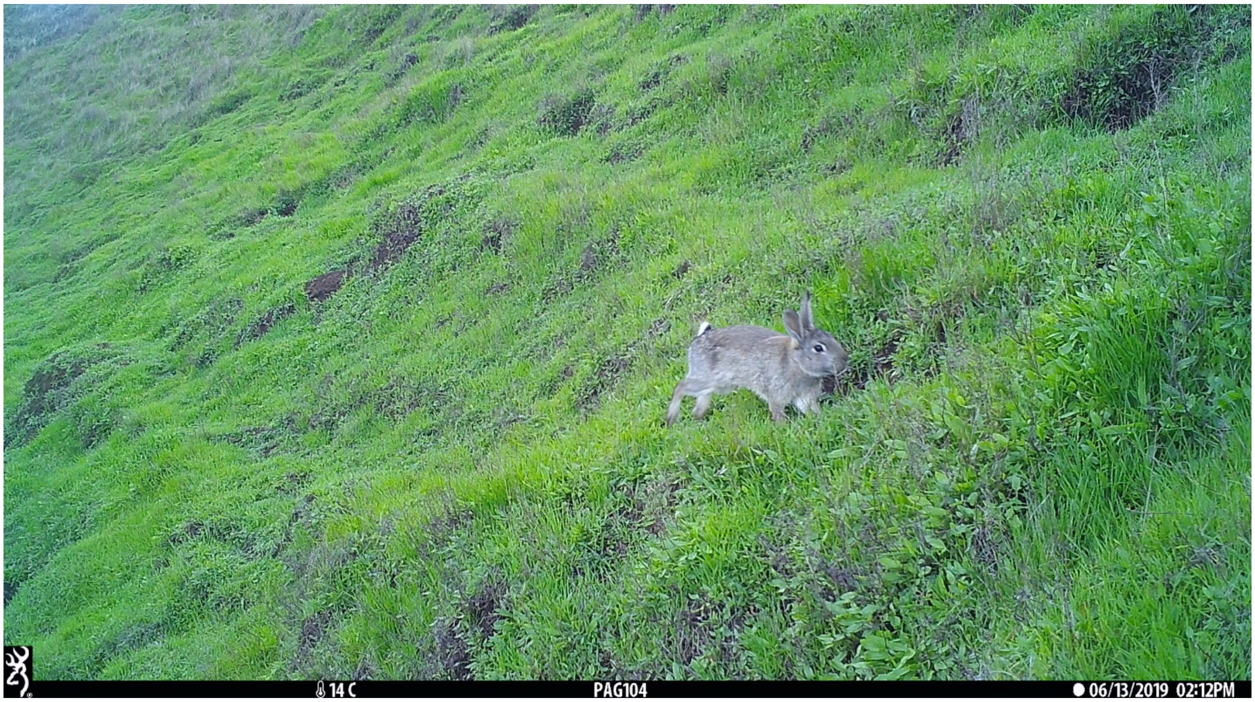


**F.** Cat (*Felis catus*) at Piedra Aguereada on IRC.

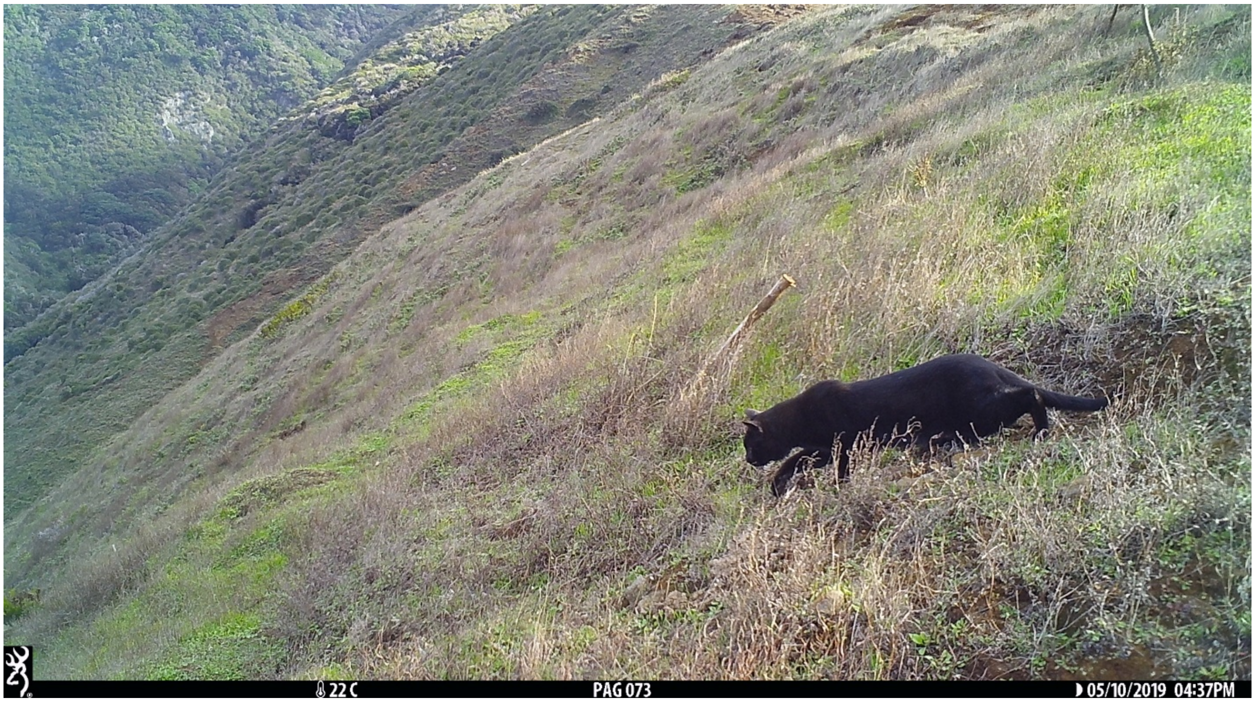

**G.** Cattle (*Bos Taurus*) at Piedra Agujereada on IRC.

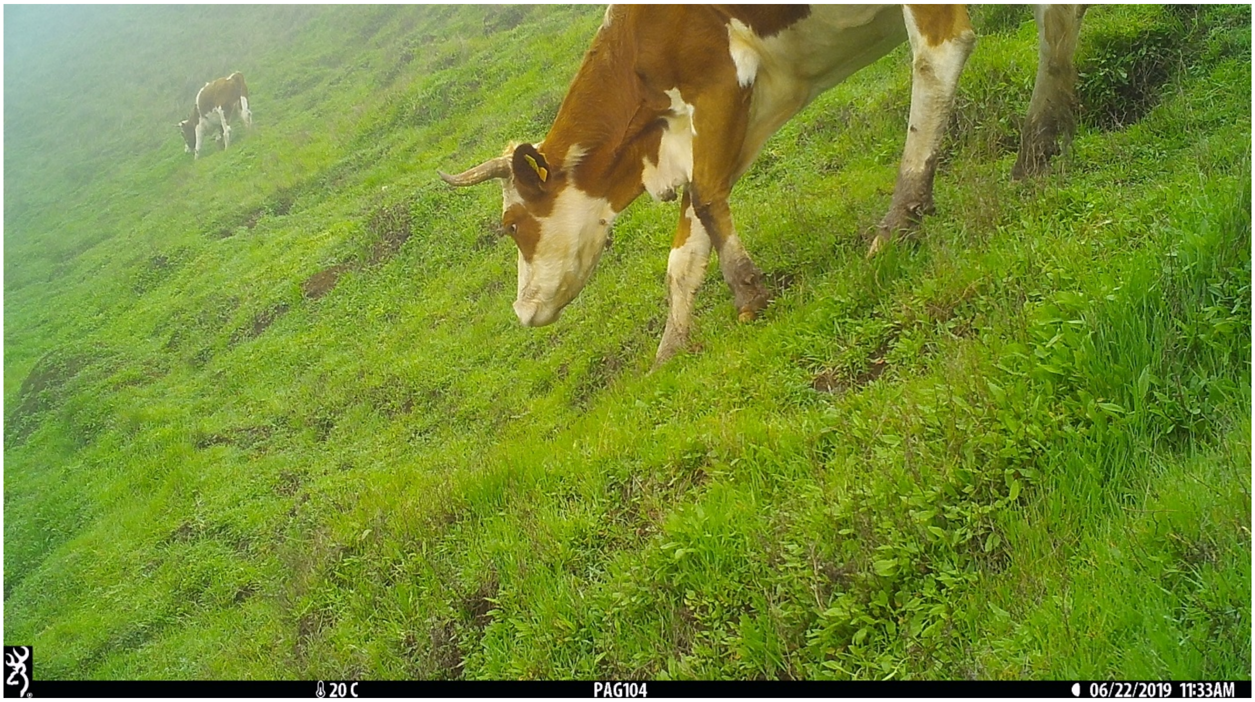


H. Rat (*Rattus* spp.; top right of image) at Piedra Agujereada on IRC.

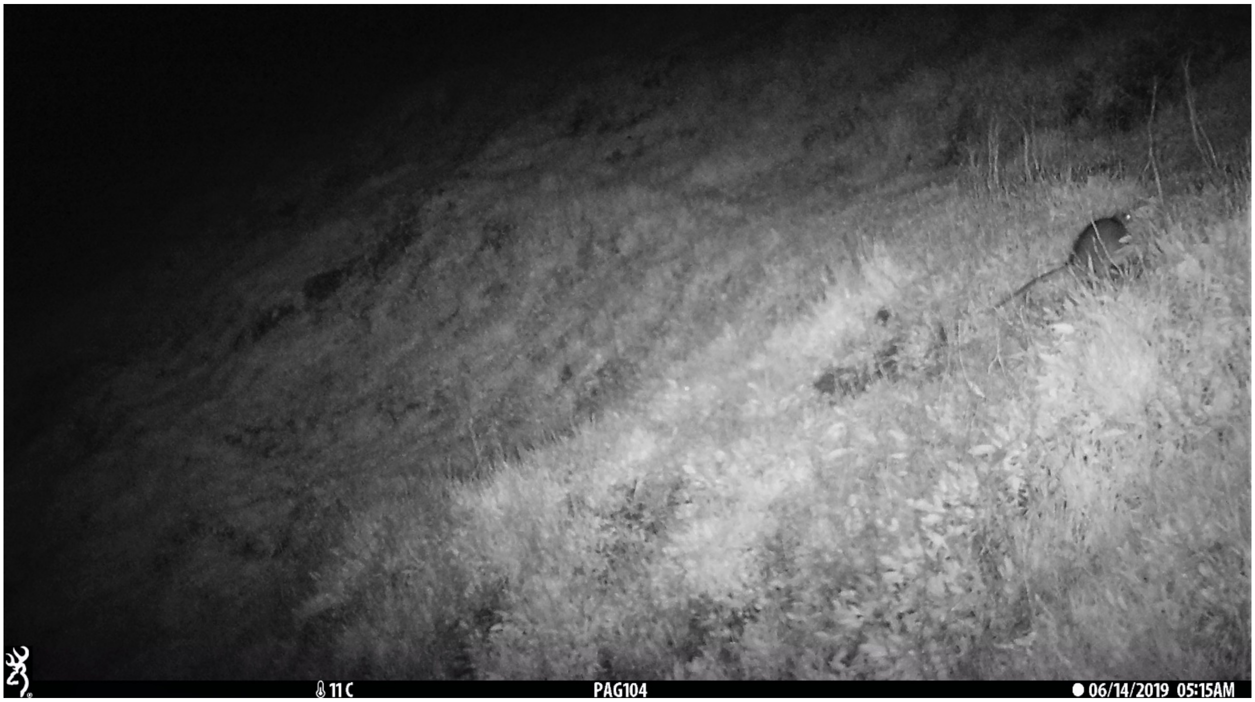

**I.** Dogs (*Canis lupus familiaris*) at Piedra Agujereada on IRC.

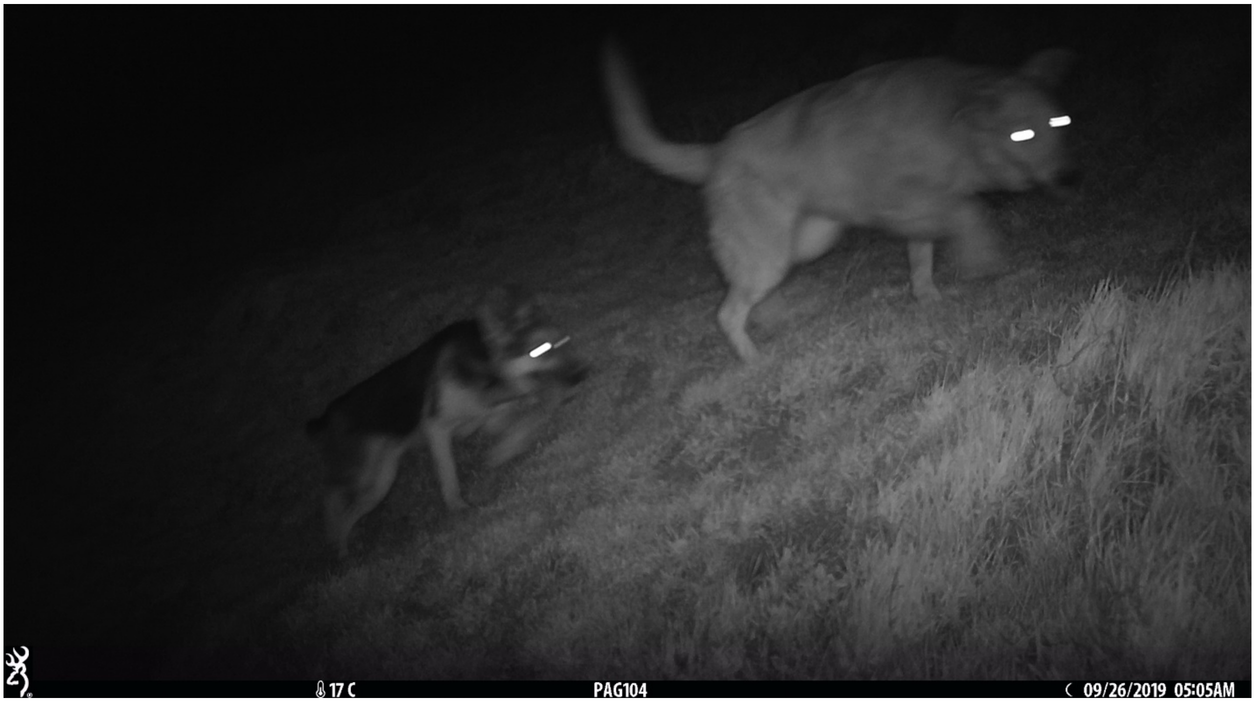


**F.** House mouse (*Mus musculus*; center of image) at Piedra Agujereada on IRC.

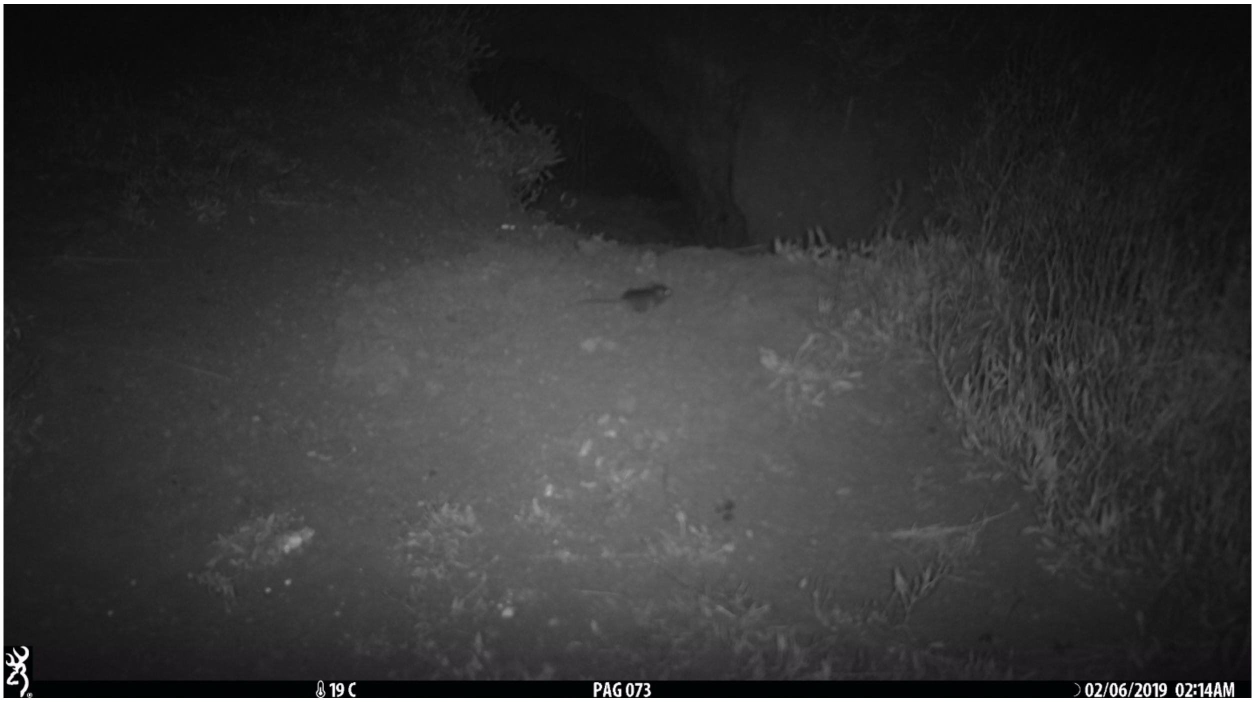

**J.** Southern coati (*Nasua nasua*) at Piedra Agujereada on IRC

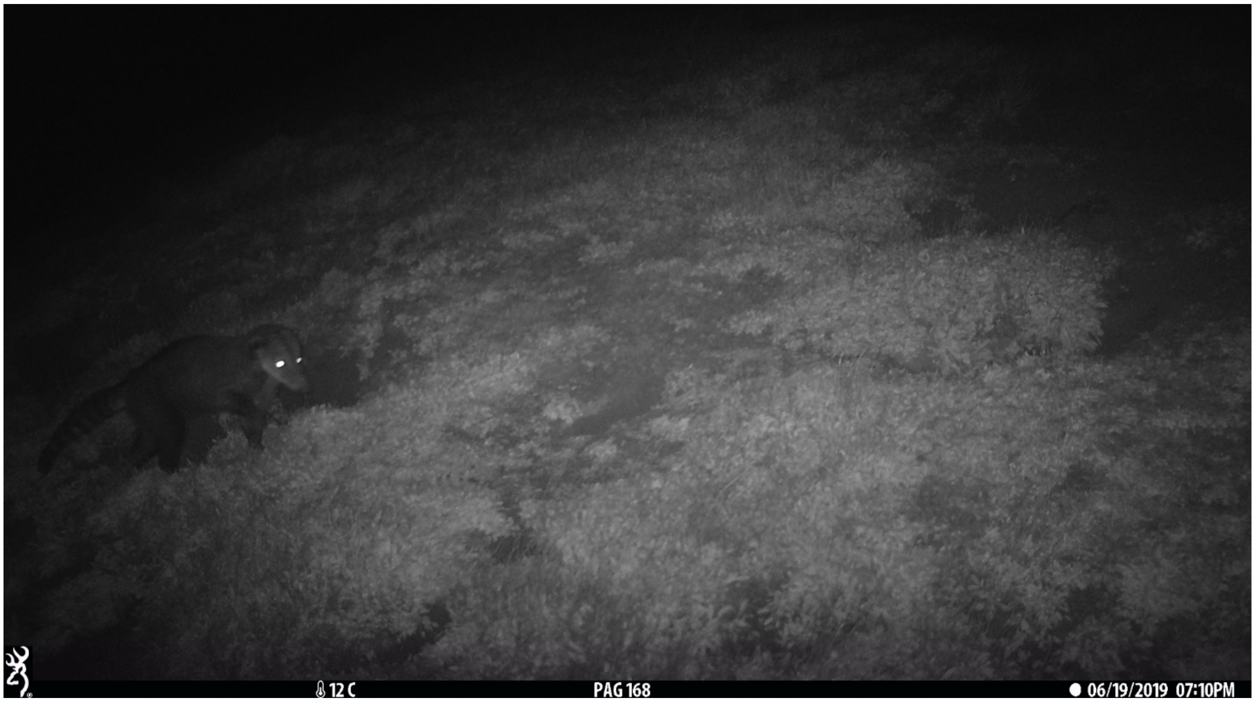

Supplement: S2 Fig — (PDF) [file pone.0254416.s005.pdf]
