# Supplementary material for: Introduced and native vertebrates in pink-footed shearwater (Ardenna creatopus) breeding colonies in Chile
Source: PLoS One. 2021 Jul 29;16(7):e0254416. doi: 10.1371/journal.pone.0254416 (PMC8321096; doi:10.1371/journal.pone.0254416)

S3 Fig. Example trail camera images of select native bird species in pink-footed shearwater (*Ardenna creatopus*) breeding colonies on Isla Mocha and Isla Robinson Crusoe (IRC), Chile.

A. Pink-footed shearwaters on Isla Mocha.

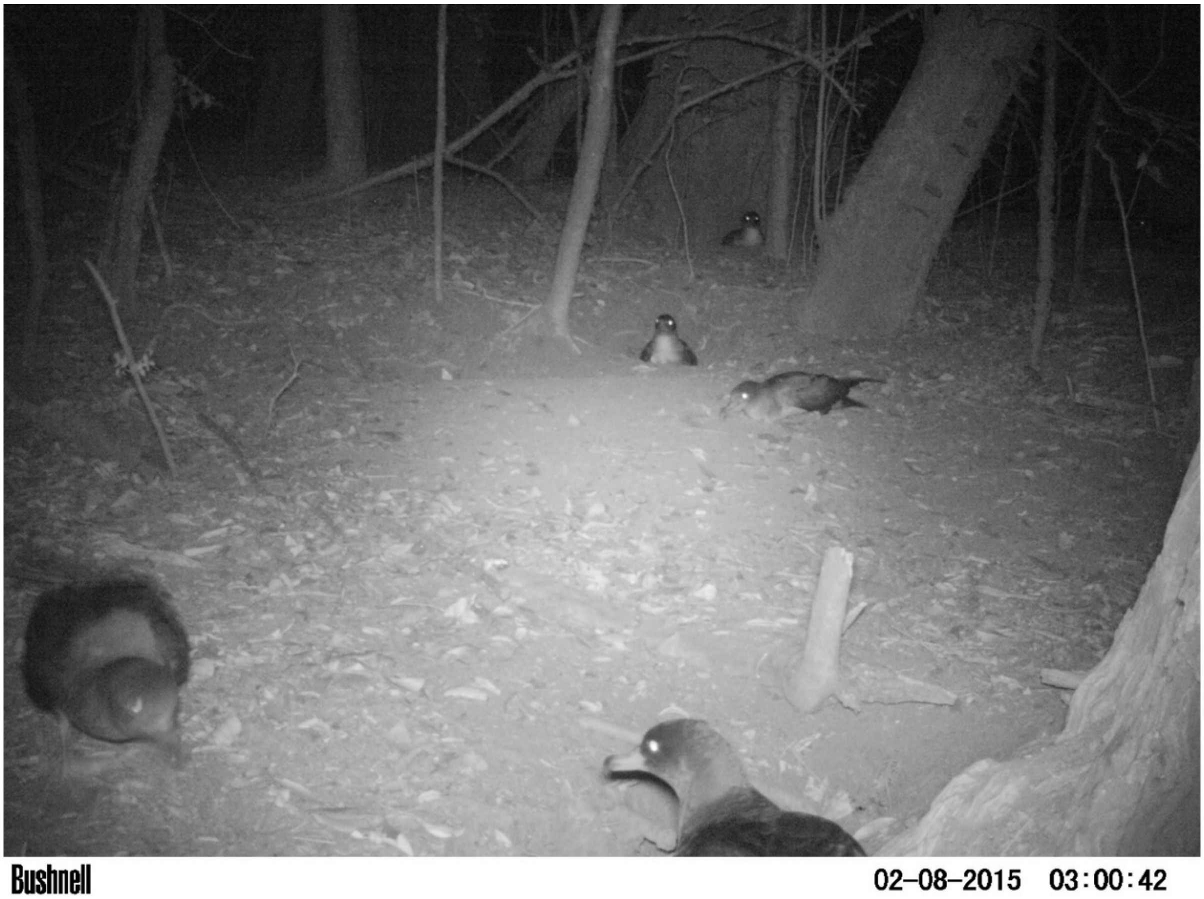

B. Mocha chucao tapaculo (*Scelorchilus rubecula mochae*) on Isla Mocha

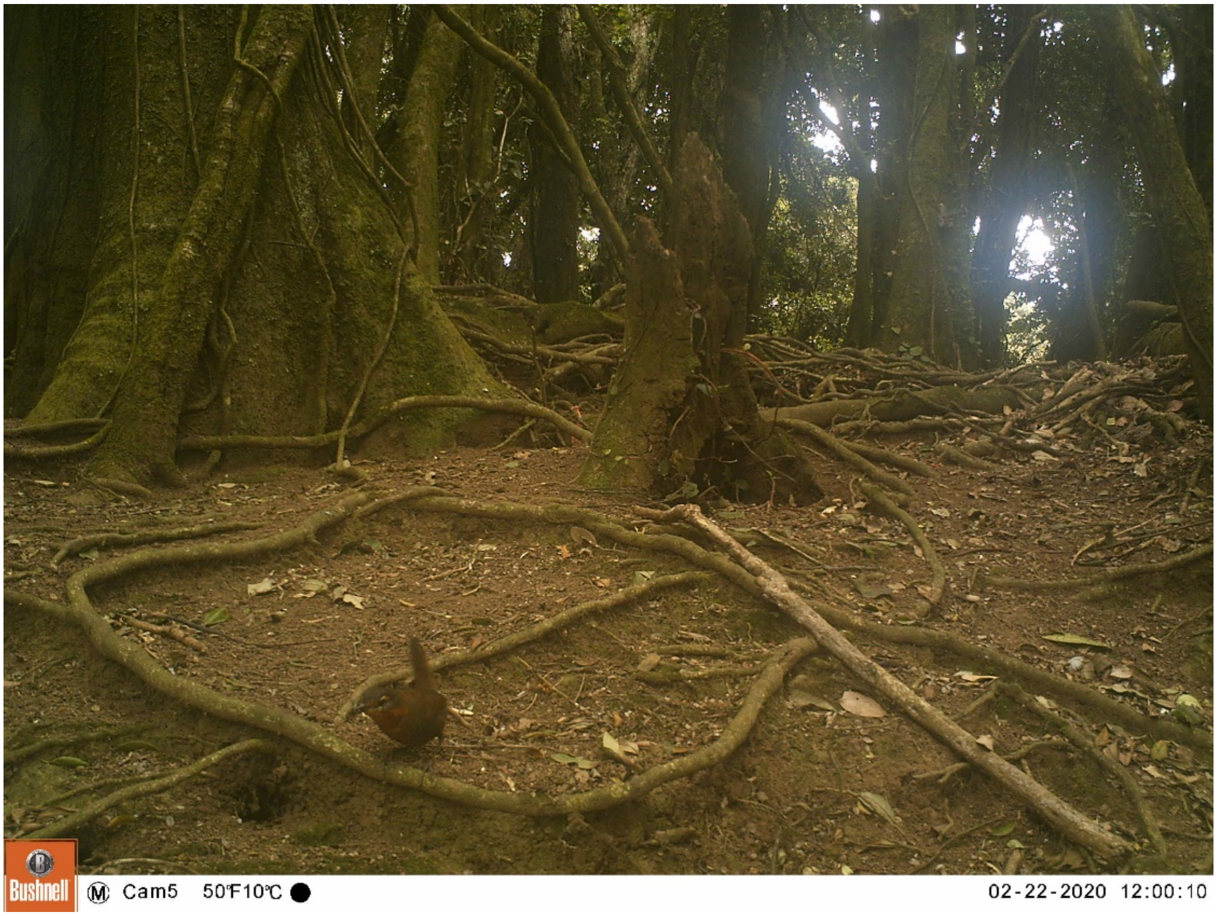

C. Austral thrush (*Turdus falklandii*) on Isla Mocha.

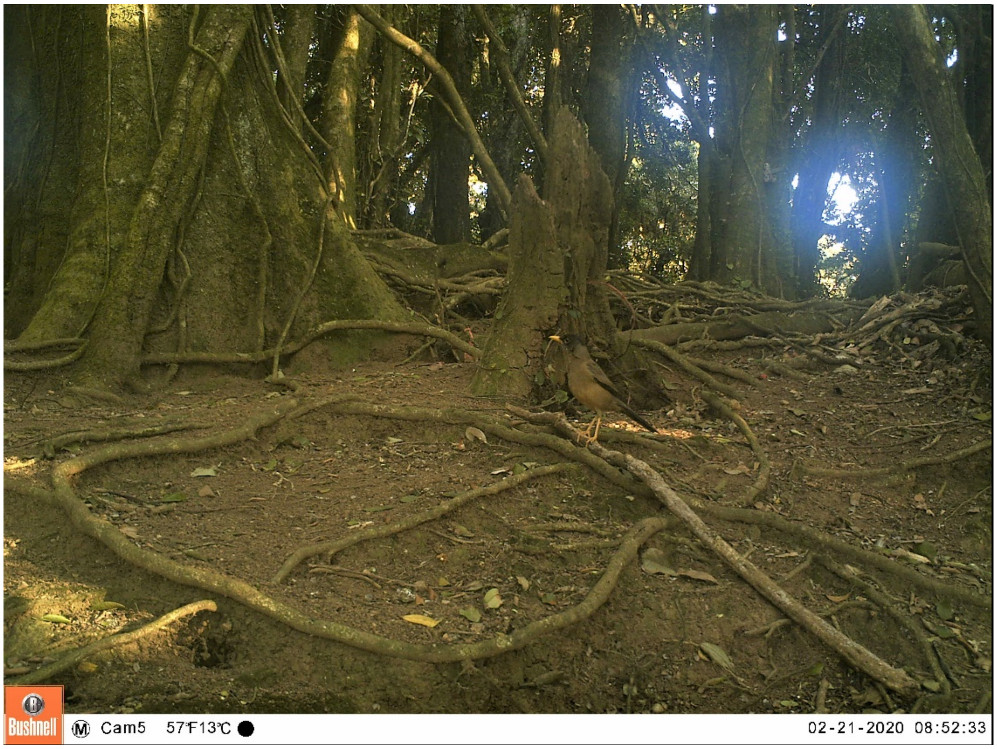

D. Juan Fernández petrel (*Pterodromoa externa*) at Piedra Agujereada on IRC.

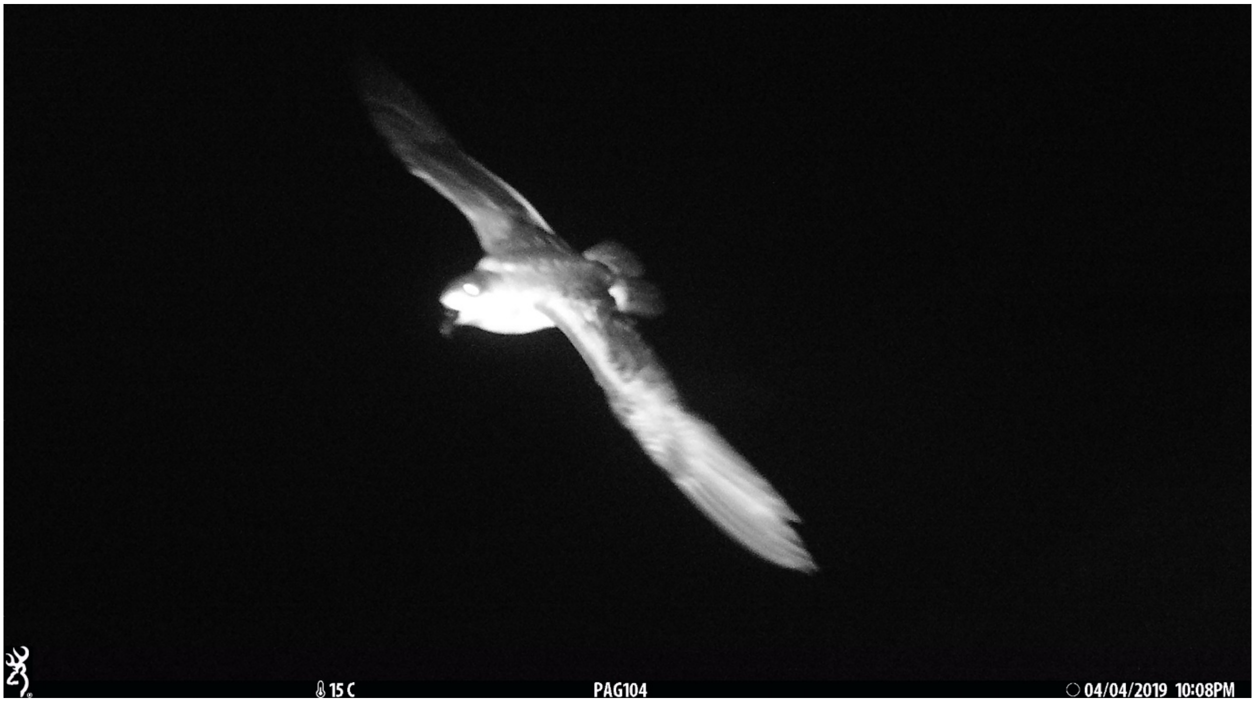

E. Juan Fernández kestrel (*Falco sparverius fernandensis*) at Piedra Agujereada on IRC.

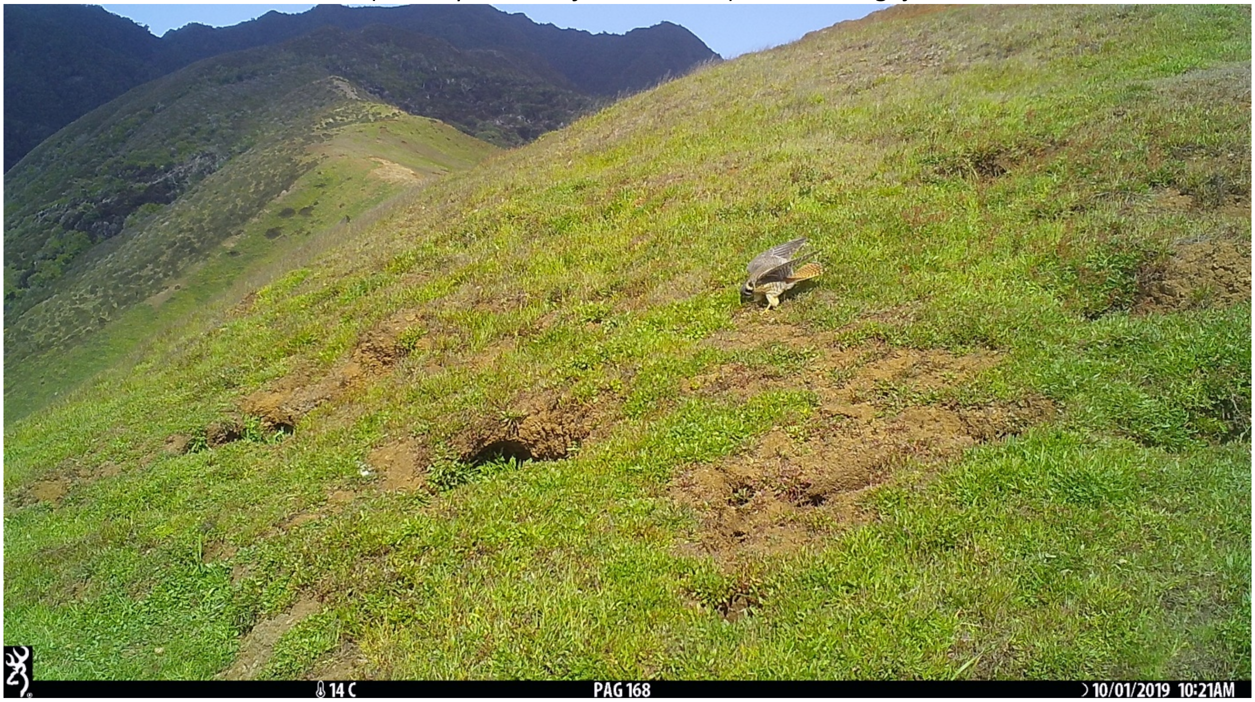

Supplement: S3 Fig — (PDF) [file pone.0254416.s006.pdf]
